# Ultrafast delocalization of excitation in synthetic light-harvesting nanorings†
†Electronic supplementary information (ESI) available: Full material synthesis and characterization. Further steady-state spectroscopy of materials. Anisotropy dynamics. Quantum yield calculations. Temperature-dependent spectroscopy. Details of Monte Carlo simulation. See DOI: 10.1039/c4sc02424a


**DOI:** 10.1039/c4sc02424a

**Published:** 2014-09-16

**Authors:** Chaw-Keong Yong, Patrick Parkinson, Dmitry V. Kondratuk, Wei-Hsin Chen, Andrew Stannard, Alex Summerfield, Johannes K. Sprafke, Melanie C. O'Sullivan, Peter H. Beton, Harry L. Anderson, Laura M. Herz

**Affiliations:** a University of Oxford , Department of Physics , Clarendon Laboratory , Parks Road , Oxford , OX1 3PU , UK . Email: l.herz@physics.ox.ac.uk; b University of Oxford , Department of Chemistry , Chemistry Research Laboratory , Oxford , OX1 3TA , UK . Email: harry.anderson@chem.ox.ac.uk; c School of Physics & Astronomy , University of Nottingham , Nottingham , NG7 2RD , UK

## Abstract

Rings of chlorophyll molecules harvest sunlight remarkably efficiently during photosynthesis in purple bacteria. The key to their efficiency lies in their highly delocalized excited states that allow for ultrafast energy migration. Here we show that a family of synthetic nanorings mimic the ultrafast energy transfer and delocalization observed in nature. π-Conjugated nanorings with diameters of up to 10 nm, consisting of up to 24 porphyrin units, are found to exhibit excitation delocalization within the first 200 fs of light absorption. Transitions from the first singlet excited state of the circular nanorings are dipole-forbidden as a result of symmetry constraints, but these selection rules can be lifted through static and dynamic distortions of the rings. The increase in the radiative emission rate in the larger nanorings correlates with an increase in static disorder expected from Monte Carlo simulations. For highly symmetric rings, the radiative rate is found to increase with increasing temperature. Although this type of thermally activated superradiance has been theoretically predicted in circular chromophore arrays, it has not previously been observed in any natural or synthetic systems. As expected, the activation energy for emission increases when a nanoring is fixed in a circular conformation by coordination to a radial template. These nanorings offer extended chromophores with high excitation delocalization that is remarkably stable against thermally induced disorder. Such findings open new opportunities for exploring coherence effects in nanometer molecular rings and for implementing these biomimetic light-harvesters in man-made devices.

## Abstract 2

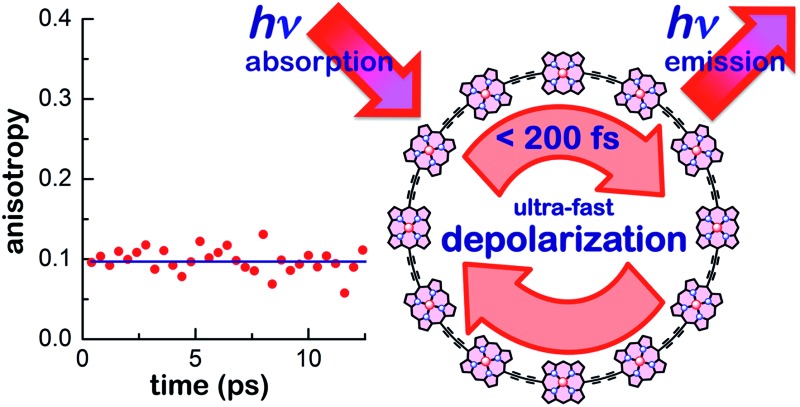
When light is absorbed by a nanoring consisting of 6–24 porphyrin units, the excitation delocalizes over the whole molecule within 200 fs. Highly symmetric nanorings exhibit thermally enhanced super-radiance.

## Introduction

Photosynthetic organisms have evolved to use a wide variety of different arrays of porphyrin derivatives (chlorophylls) to harvest sunlight.^1–4^ These pigment assemblies often support highly delocalized excited states to facilitate the transfer of energy between adjacent antennae, such as those in the light harvesting complex 2 (LH2) of bacterium *Rhodobacter sphaeroides*.^5–9^ It has recently been suggested that in typical organic semiconductors designed for photovoltaic devices, the existence of delocalized states may similarly aid charge-separation following light absorption.^10^ Materials that support delocalized electronic wavefunctions as a consequence of their structural rigidity (such as fullerenes and porphyrins), are expected to make excellent components for next-generation carbon-based solar cells.^11,12^ The designs that nature has invented provide tremendous inspiration for the creation of new molecular light-harvesting materials. Many chromophore arrays have been synthesized in an attempt to mimic LH2, but most of them exhibit incoherent exciton hopping.^13–17^ Here we demonstrate that ultrafast excited-state delocalization can be achieved in large arrays if there is sufficient electronic coupling between the component subunits and sufficient structural order.

A peculiar consequence of the high symmetry of natural chlorophyll ring assemblies (such as the B850 complex in LH2) is that transitions to their lowest exciton states are dipole-forbidden.^18^ However, distortions and disorder allow a partial transfer of oscillator strength from higher-lying allowed transitions.^18–20^ These mechanism ought to allow thermally activated distortions to cause superradiance with increasing temperature.^1^ Such effects have so far not been observed for natural systems, because the resulting vibrations also electronically decouple the individual chlorophyll chromophores, leading to a breakdown of exciton delocalization.^20,21^ π-Conjugated chromophore arrays are expected to be less susceptible to such electronic decoupling, yet thermally enhanced emission has to date only been demonstrated for small molecules such as benzene,^22,23^ where it arises from dynamic symmetry breaking (Herzberg–Teller). Recent single-molecule experiments on nanorings synthesized from phenylcarbazole units linked with phenylene–ethynylene–butadiynylene groups have shown that structural relaxation following excitation induces exciton localization on segments of the π-system.^24^ As a result, these nanorings are highly emissive, showing no evidence of a delocalized, dipole-forbidden excited state. These results suggest that if electronic delocalization is to be maintained in the excited state, structurally rigid chromophores have to be selected, with porphyrin derivatives being a natural choice.

**Fig. 1:**
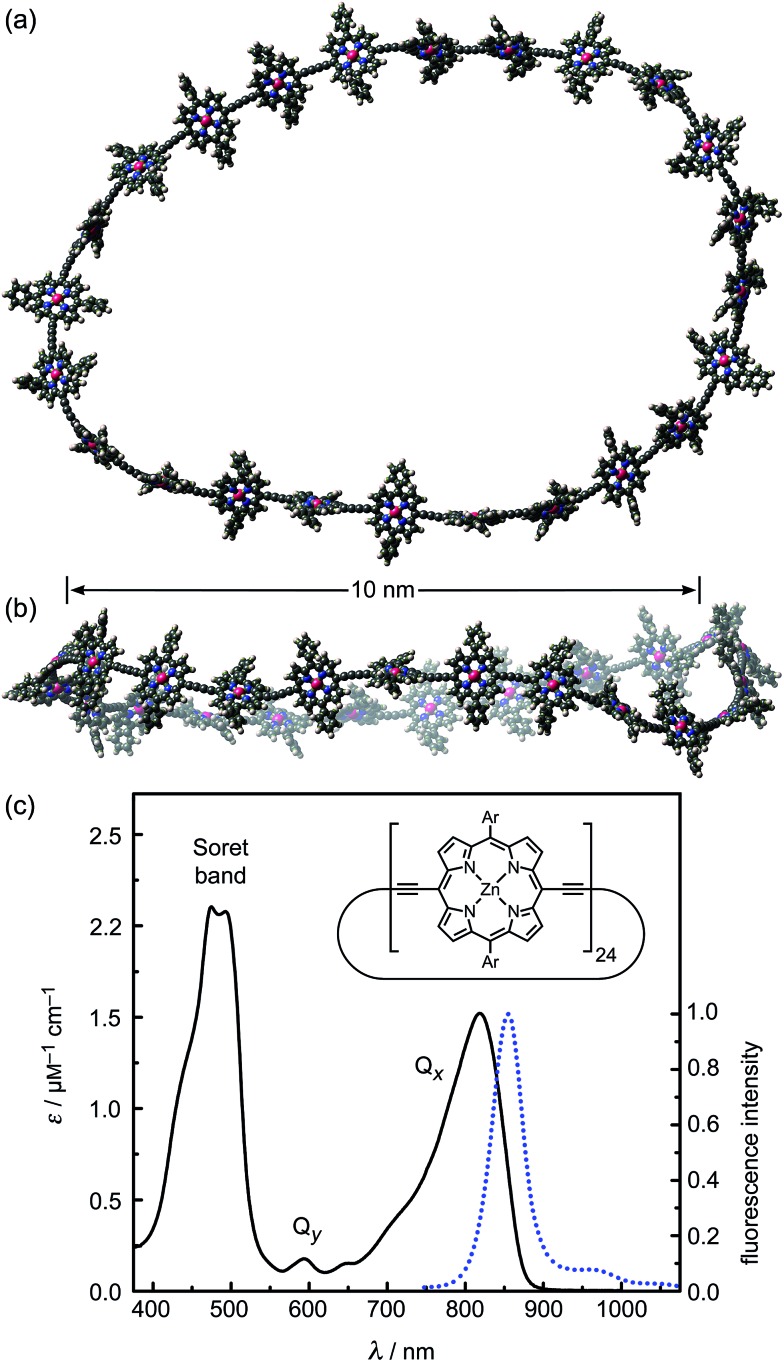
(a and b) Two orthogonal views of a snapshot of ***c*-P24** from a molecular dynamics simulation at 300 K (using the MM+ force field implemented in HyperChem). (c) Molar extinction (black line) and normalized photoluminescence (blue dashed line) spectra at 295 K of ***c*-P24** cyclic porphyrins in toluene/1% pyridine. The emission was recorded after excitation at 770 nm (1.61 eV). The insert shows the chemical structure of ***c*-P24** (Ar = 3,5-bisoctyloxyphenyl).

In this study we show that a family of π-conjugated porphyrin nanorings exhibit ultrafast excitation delocalization around the ring following light absorption. These porphyrin nanorings have become accessible due to recent advances in template-directed synthesis.^25–29^ Remarkably, we find that the excited electron–hole wavepacket delocalizes rapidly around the ring (within less than 200 fs), even for the largest ring containing 24 porphyrin units (diameters of 10 nm, see Fig. 1). For smaller rings, the lowest emitting transitions of the nanorings are shown to be dipole-forbidden as a result of the high symmetry, showing that significant wavefunction delocalization is maintained in the emitting state. We demonstrate that such symmetry constraints may be lifted through both static and dynamic distortions of the rings, and that thermally activated distortions substantially enhance the emission rate. These synthetic porphyrin nanorings exhibit electronic wavefunction delocalization similar to that found in the most strongly coupled biological light-harvesting systems, such as the B850 ring in LH2.^7^

## Experimental

### Synthesis

The synthesis, purification and characterization of the cyclic and linear porphyrin compounds were carried out according to previously described procedures,^25–29^ as detailed in the ESI.†

### Time-resolved photoluminescence spectroscopy

The photoluminescence up-conversion technique was used to detect the photoluminescence (PL, fluorescence) emitted from solutions (10^–3^ to 10^–4^ M) held in a quartz cuvette. Samples were excited with the output of a mode-locked Ti:sapphire laser providing pulses of 100 fs duration at a photon energy of 1.61 eV (770 nm) or 1.51 eV (820 nm) and a repetition rate of 80 MHz. The emerging photoluminescence was collected in a front-excitation, front-emission mode and optically gated in a beta-barium oxide (BBO) crystal by a time-delayed linearly polarized gate pulse. The resulting sum-frequency photons were collected, dispersed in a monochromator and detected by a liquid nitrogen cooled CCD. The polarization of the excitation beam (with respect to the polarization of the detected PL) was varied through rotation of a half-wave plate and a Glan–Thompson polarizer. The PL anisotropy *γ* was calculated from the PL intensity components polarized parallel (*I*_para_) and perpendicular (*I*_perp_) to the polarization of the excitation pulse, using:

1 *γ* = (*I*_para_ – *I*_perp_)/(*I*_para_ + 2*I*_perp_)

Frontal excitation was associated with an excitation–gate cross-correlation width of 200 fs resolution for surface scatter. Imaging effects in solution led to an additional rise tail of a few hundred femtoseconds in the gated PL components, however this had an insignificant effect on the derived PL anisotropy. For longer (>1 ns) time delays the PL decay dynamics were resolved using electronic gating through time-correlated single-photon counting (Becker & Hickl GmbH) with a temporal resolution of 180 ps and detection based on a Peltier-cooled photomultiplier tube.

### Temperature-dependent, time-integrated PL spectroscopy

Cuvettes holding solution samples were placed in nitrogen exchange gas, inside a liquid-nitrogen cryostat controlled by a heater and a thermocouple. To avoid freezing and evaporation of solution, the temperature range was limited to 220–360 K. Samples were excited under the same conditions as described above and *I*_para_ was detected using the same spectrometer and CCD, but replacing the BBO crystal with a Glan–Thompson polarizer. To detect and correct for thermal drifts in the spectroscopic detection system, a silicon reference diode of known spectral response, referenced to the now chopped excitation (329 Hz) beam using a lock-in amplifier, was placed nearby the sample emission. The measured PL spectra were corrected for instrumental response with a tungsten filament lamp of known emissivity.

## Results and discussion

### Steady-state fluorescence

**Fig. 2:**
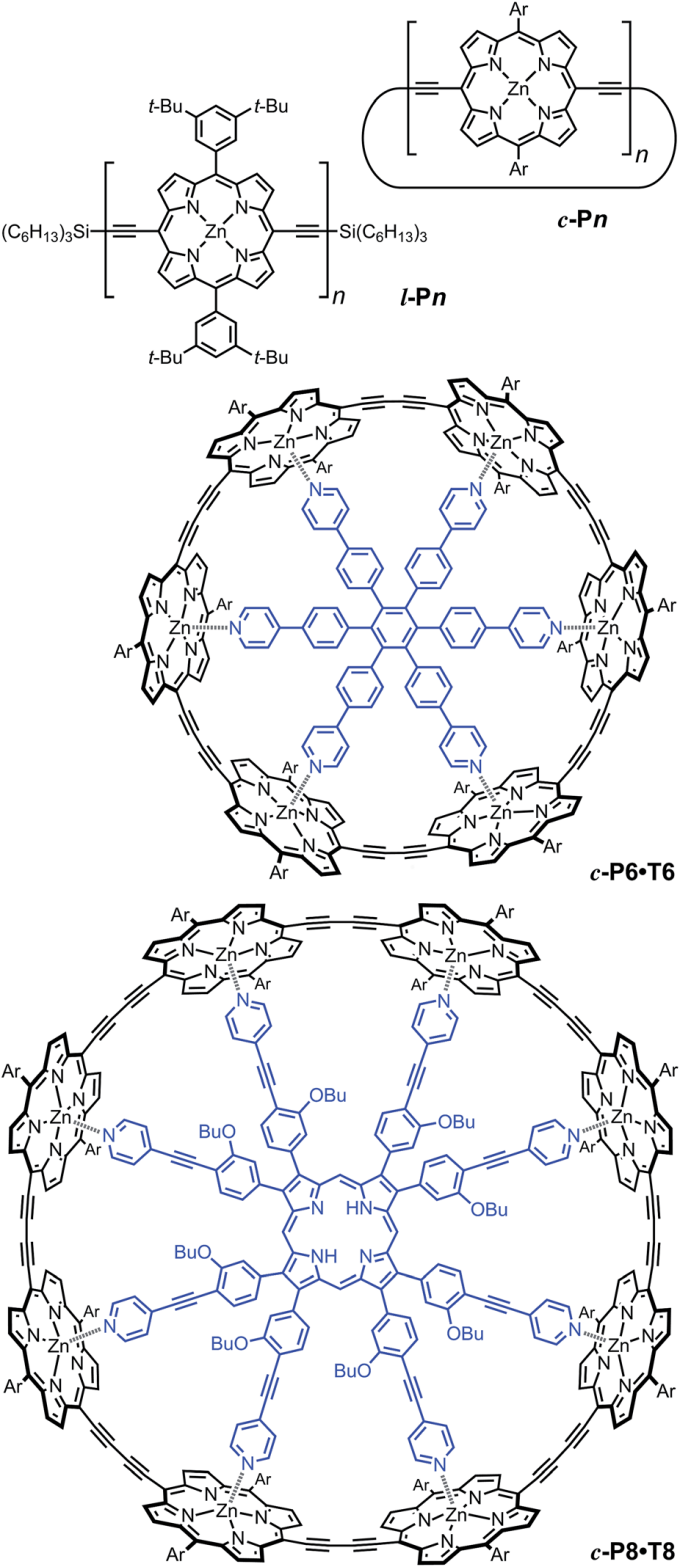
Structures of cyclic oligomers ***c*-P*n***, linear oligomers ***l*-P*n***, the 6-porphyrin nanoring template complex ***c*-P6·T6** and the 8-porphyrin nanoring template complex ***c*-P8·T8**. Template units are shown in blue. “Ar” indicated 3,5-di(*tert*-butyl)phenyl.

**Fig. 3:**
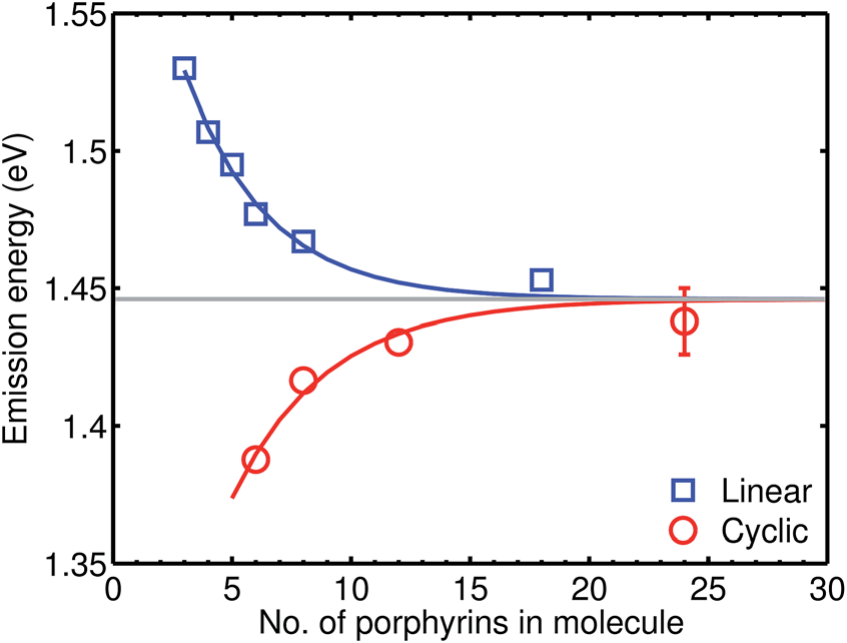
Plot of the photon energy of the peak *Q*_*x*_ PL band for linear oligomers (***l*-P*n***, *n* = 3, 4, 5, 6, 8 and 18; blue curve) and cyclic oligomers (***c*-P*n***, *n* = 6, 8, 12 and 24; red curve) recorded in toluene/1% pyridine at 295 K against the number of porphyrin units in the oligomer (*n*). The solid lines are fits according to eqn (2), while the error bar represents the peak fitting uncertainty for all samples.

We examine a family of porphyrin nanorings consisting of 6, 8, 12 or 24 porphyrin units linked by butadiyne bridges, as shown in Fig. 2. The largest member of this family, ***c*-P24**, has a diameter of 10 nm (Fig. 1), which is significantly larger than the chlorophyll ring in LH2 (ref. 2) and slightly larger than that in LH1 found in purple bacteria.^30^ Insights into the electronic structures of these nanorings can be gained by comparing their absorption and photoluminescence (PL, fluorescence) spectra with those of the corresponding linear oligomers ***l*-P*n***. Fig. 1c shows the steady-state absorption and time-integrated PL spectra of ***c*-P24** in solution; spectra for the full series of rings ***c*-P*n*** and linear chains ***l*-P*n*** are shown in the ESI.† Typically for these chromophores,^31,32^ π-conjugation through the butadiyne link lifts the degeneracy of the lowest-energy Q-band of the porphyrin monomer into transitions polarized parallel (Q_*x*_) and perpendicular (Q_*y*_) to the C≡C axis.^33^ The photon energy of the Q_*x*_ PL band maxima (*E*_*n*_) changes as a function of the number of porphyrin units in the oligomer (*n*) as shown in Fig. 3. The data fit well to Meier's model, eqn (2), where *E*_∞_, *E*_1_ and *a* are empirical parameters.^34^

2 *E*_*n*_ = *E*_∞_ + (*E*_1_ – *E*_∞_)e^–*a*(*n*–1)^

The PL bands of the linear oligomers ***l*-P*n*** shift to lower energy with increasing chain length, like most π-conjugated oligomers, indicating efficient electronic coupling between the individual porphyrin units.^35,36^ Surprisingly, the PL bands of the cyclic oligomers ***c*-P*n*** shift in the opposite direction, with the same asymptote *E*_∞_. Counterintuitive behavior of this type has been reported in cycloparaphenylenes (CPPs), from both experimental^37,38^ and computational^39,40^ studies, and has been attributed to coupling of electronic transitions with static and dynamic distortions.^40^ In the smaller CPPs, geometrical distortion of the p-phenylene π-system appears to contribute to the blue-shifted PL,^41^ whereas there is very little distortion of the porphyrin π-system in ***c*-P6**, and the blue-shifted PL arises from Herzberg–Teller vibronic coupling.^28^ We note that the emission energies of cyclic and linear oligomers approach each other for large *n*, indicating that Herzberg–Teller coupling is less important for large nanorings. The blue-shift with increasing ring size shown in Fig. 3 is completely different from the behavior exhibited by other cyclic porphyrin oligomers that have been tested as LH2 mimics, which exhibit PL spectra only slight shifted from those of the component monomer or dimer unit.^14–17,42^

### Time-resolved photoluminescence anisotropy

**Fig. 4:**
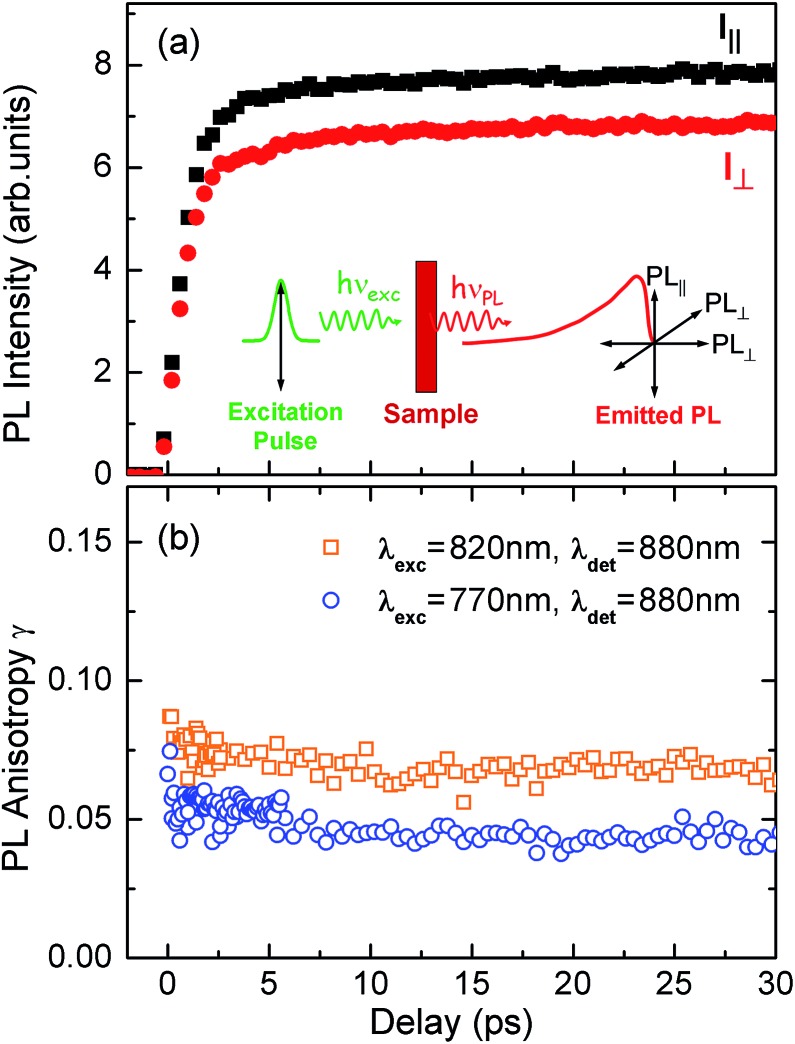
(a) PL emission transients of ***c*-P24** in toluene/1% pyridine for excitation at a photon wavelength of 770 nm and detection at 880 nm. Solution samples were excited with pulse polarization either parallel (*I*_para_, black squares) or perpendicular (*I*_perp_, red circles) to the detection polarization, as illustrated in the inset. (b) The derived PL polarization anisotropy *γ*, defined by eqn (1) as a function of time after excitation at 770 nm and 820 nm, for detection at 880 nm. Measurements with excitation at 770 nm and detection at 870 nm give identical results to those with excitation at 770 nm and detection at 880 nm.

To test the dynamics of exciton delocalization following photoexcitation of the porphyrin nanorings, we performed polarization-sensitive ultrafast time-resolved PL measurements. Solutions of nanorings were excited by 100 fs laser pulses at a wavelength of 770 nm and the PL dynamics were monitored at the emission peak energy for nanorings of a given size. The excitation fluence was kept low (0.15 μJ cm^–2^) in order to prevent PL quenching *via* exciton–exciton annihilation.^43,44^ Fig. 4a shows the transient PL of ***c*-P24** nanorings probed at the emission wavelength of 880 nm. The samples were photoexcited with pulse polarization either parallel or perpendicular to the detection polarization, as illustrated in the inset of Fig. 4a. From these traces, we extracted the time-dependent PL emission anisotropy *γ*, defined by eqn (1).

For a rigid, linear butadiyne-linked porphyrin oligomer, selective excitation near the *Q*_*x*_-band will create an excited state with a well-defined transition dipole moment oriented along the long axis of the molecule.^32,33,45^ For random distributions of such linear molecules in solution, a polarized excitation will hence preferentially excite molecules oriented in the direction of this polarization, resulting in an initial PL anisotropy *γ*(0) of 0.4, in the absence of any depolarization. However, *γ*(0) = 0.1 can be expected when full polarization memory loss occurs in the 2D plane.^46^ We excite into higher-lying allowed excited states and monitor the emission from an energetically relaxed lower state, hence we do not expect to observe effects arising from coherent coupling of orthogonal transition dipoles. If the photogenerated exciton delocalized in the plane of a flat nanoring within the timescale of the excitation pulse, we would expect an initial anisotropy of *γ*(0) = 0.1. However, additional depolarization may arise, *e.g.* by out-of-plane ring distortions which will further lower the value of *γ*. Fig. 4b shows the evolution of *γ* as a function of time delay after excitation for ***c*-P24** (curves for all other compounds are given in ESI†). We find that for all porphyrin nanorings and linear oligomers under investigation, *γ* is constant within the first 30 ps after excitation, indicating that slower effects such as molecular re-orientation are absent. For all the nanorings investigated here, any initial depolarization is faster than the time-resolution of our technique, which is about 200 fs. We find that for the largest nanoring in the series, *γ* depends on the excitation energy but not the emission energy. Exciting ***c*-P24** at the high-energy edge (770 nm) of the *Q*_*x*_-band absorption results in a relatively low value of *γ*(0) = 0.06 suggesting that significant out-of-plane distortions are possible and that these distorted conformers have blue-shifted absorption. Exciting nearer to the red edge of the absorption spectrum at a wavelength of 820 nm, we find *γ*(0) = 0.08, a recovery toward the value expected for full excitation delocalization on planar rings. These measurements therefore suggest that for the largest ***c*-P24** rings, a range of conformations are present in the ensemble, as expected from molecular dynamics calculations (Fig. 1a and b).

**Fig. 5:**
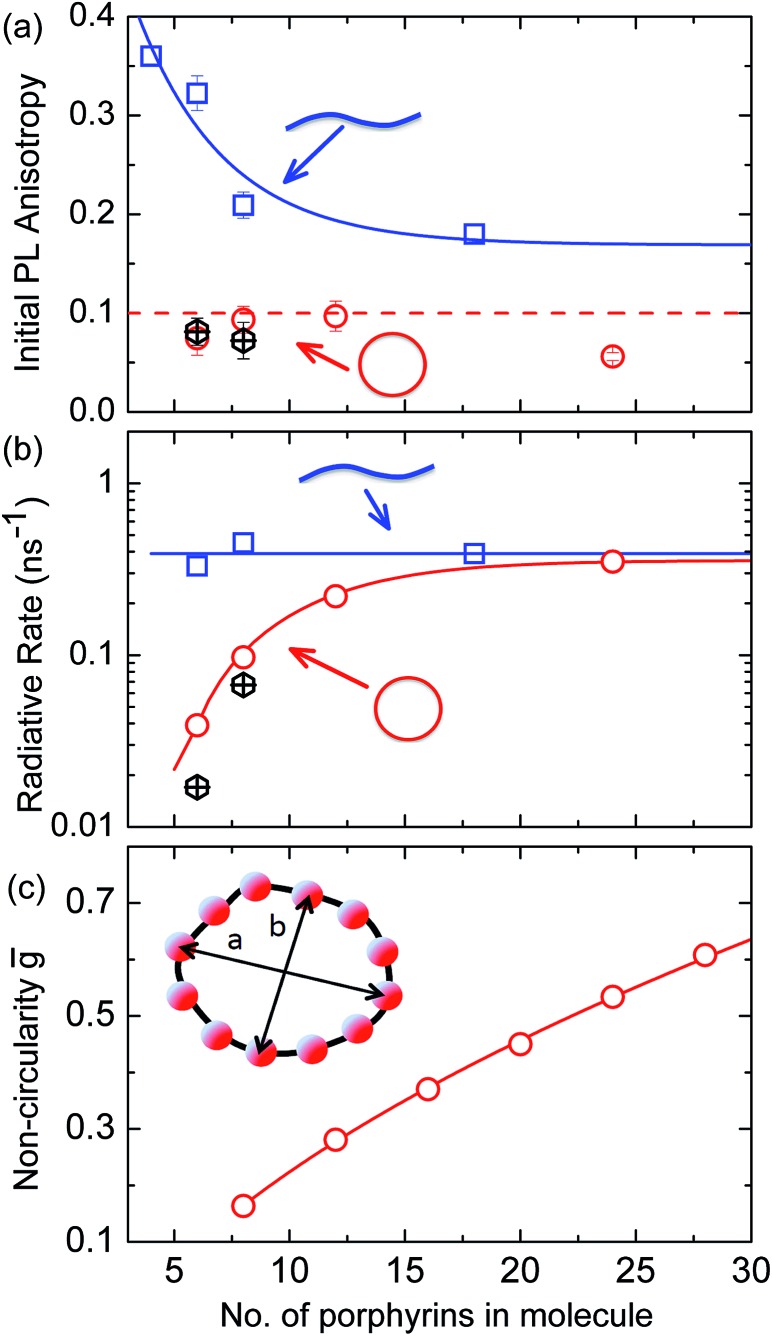
(a) Initial PL polarization anisotropy within the first few hundred femtoseconds after excitation at 770 nm, as a function of the number of porphyrins contained in the molecule, for free nanorings (red open circles), template-bound nanorings (black crossed hexagons) and linear oligomers (blue open squares) in solution. (b) Radiative transition rate as a function of the number of porphyrins. The transition rates for free porphyrin nanorings, template-bound porphyrin nanorings and porphyrin linear oligomers are represented by the red open circles, black crossed hexagons and blue open squares, respectively. (c) Ensemble-averaged deviation 
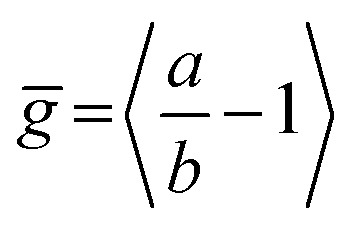
 of nanorings from circularity, where *a* and *b* are the long and short axes of each nanoring. was determined from Monte Carlo modeling at 290 K as described in the main text, using parameters defined by correlation with conformations observed in STM images. All lines are guides to the eye.

More generally, we find that complete polarization memory loss in the ring plane is observed for nanorings incorporating between 6 and 24 porphyrin units. Fig. 5a shows that the initial PL anisotropy measured within the first few hundred femtoseconds after excitation is just below 0.1 for the smaller nanorings and drops to 0.06 for the largest (***c*-P24**), suggesting that larger rings are more susceptible to deformations. To rule out the presence of experimental artifacts, we also measured the PL depolarization for matching linear porphyrin oligomers, ***l*-P*n***, for *n* = 4, 6, 8, 18 and 26 (see ESI†). As expected, linear oligomers retain a significantly higher fraction of their polarization memory than nanorings with the same number of porphyrin units. The observed small loss of PL anisotropy below the value of 0.4 expected for a rigid linear transition dipole has been attributed to the worm-like nature of the longer linear oligomers,^32^ in agreement with high initial excitonic wavefunction delocalization for oligomers incorporating up to eight porphyrins.

The observed ultrafast polarization memory loss in the ring plane for porphyrin nanorings up to even the largest ***c*-P24** rings is remarkable given the extended ring diameters of up to 10 nm. Such ultrafast exciton delocalization may derive from a variety of mechanisms. On the one hand, absorption into a fully delocalized state, for which the highest occupied and lowest unoccupied molecular orbitals are spread over the whole ring, would lead to an emitting transition moment whose orientation has no correlation with the polarization of the excitation pulse. On the other hand, the ultrafast emission depolarization may originate from an excited state whose electron–hole wavepacket is much more localized but moves rapidly (and possibly coherently) around the ring on the 100 fs timescale. This scenario may also be viewed as an initial generation of a coherent superposition of localized adiabatic states that dephase into a localized state on an ultrafast timescale. While our ultrafast depolarization measurements alone cannot distinguish between these two cases, our analysis of the radiative transition rates and temperature-activated emission (see below) suggests that a gradual shift from the former to the latter scenario occurs as the rings increase in size.

### Radiative rates

To examine the subsequent evolution of the emitting transition dipole moment on the nanorings, we determined their radiative rates as a function of ring size (Fig. 5b), as derived from PL intensity transients and quantum yields (see ESI†). A steep increase in radiative rate of almost two orders of magnitude is observed when the nanoring size is increased from 6 to 24 porphyrin units. For small porphyrin oligomers (*n* = 6, 8), the circular molecules have radiative rates that are 1–2 orders of magnitude lower than those of their linear counterparts. These results are reminiscent of the situation for small ring-like molecules such as benzene^22,23,49^ and porphyrin assemblies in natural light-harvesting systems^3,19^ for which the symmetry of the ring yields a lowest excited state that is all but dipole-forbidden. It appears from our measurements of the radiative rate of the emitting state (Fig. 5b), that such symmetry constraints are predominantly lifted for the large (*n* = 24) porphyrin nanoring. A key to the mechanisms behind this effect is provided by comparing the *n* = 6 and 8 nanorings into which a rigidifying template has been inserted to form the complexes ***c*-P6·T6** (ref. 27 and 28) and ***c*-P8·T8** (ref. 29) (Fig. 2). Fig. 5b shows that rings with freedom to distort have significantly higher emission rates than identical rings that are constrained by a template into a more rigid geometry. Hence deviations from ring-like symmetry are most likely to cause the recovery in emission dipole strength in the larger rings.

**Fig. 6:**
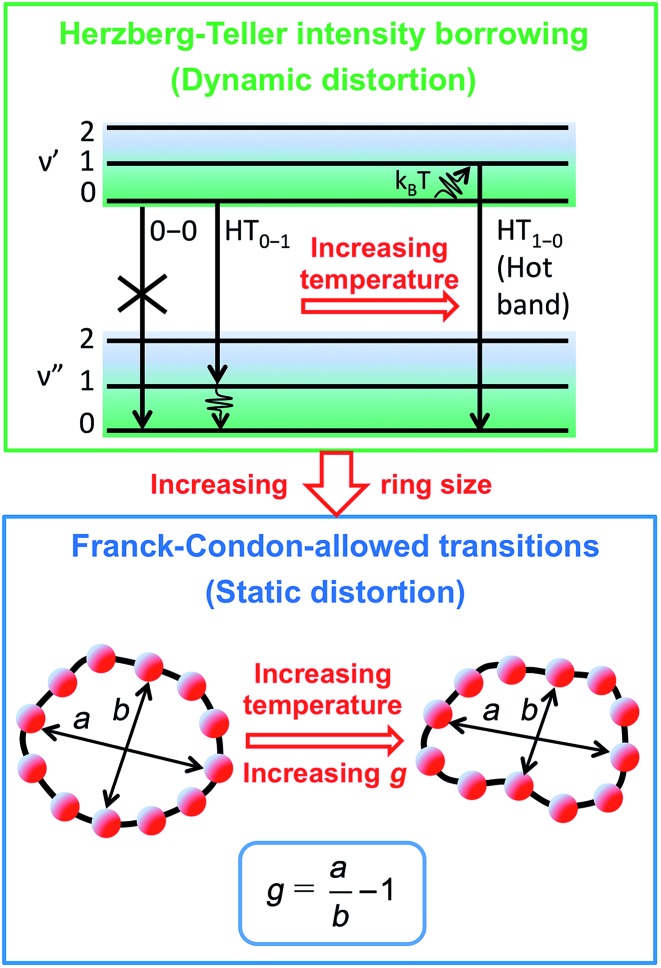
Schematic diagram illustrating the processes leading to coupled electronic-vibrational transitions in porphyrin nanorings. Large rings are more flexible, and hence static distortions from symmetry yield lowest vibronic transitions that are allowed within the standard Franck–Condon approximation. For small rings, Herzberg–Teller intensity borrowing from higher-lying allowed transitions yields emission from the lowest state through involvement of a non-totally symmetric vibration quantum.


Such distortions can be of a simple static nature, *i.e.* recombination occurs from a ring whose geometry deviates from circularity. Alternatively, they may be considered to derive from dynamic effects, such as coupling with suitable vibrations that may induce dynamic self-localization or intensity borrowing from higher-lying states of different symmetry. Fig. 6 gives a diagrammatic representation of the impact of these two mechanisms on the strength of the electronic transition moment. In most cases, polyatomic molecules can be well described under the Franck–Condon (FC) principle, assuming that electronic transitions take place much more rapidly than the accompanying shifts in nuclear positions.^50,51^ Under such approximations, the overall moment for the coupled vibronic transition may be factorized into its electronic part, *R*_e_ and its vibrational overlap integral. For molecules with certain high symmetries, such as benzene,^49^ or Buckminster fullerene (C_60_),^52^ the lowest electronic transition becomes dipole-forbidden, (*R*_e_)_eq._ ≈ 0. However, some emission may be observed through Herzberg–Teller (HT) intensity borrowing, by involving a non-totally symmetric vibration in a coupled electronic-vibrational transition. As a result, intensity can be “borrowed” from a higher-lying electronically allowed state of symmetry that matches the coupled symmetry of the lowest state and the involved vibration.^50,51^ Such HT transitions may be understood in terms of a breakdown of the FC approximation allowing the electronic transition moment *R*_e_ to be expanded into a Taylor series in the normal coordinates *Q*_*k*_ associated with the relevant vibrations:^50,51^

3

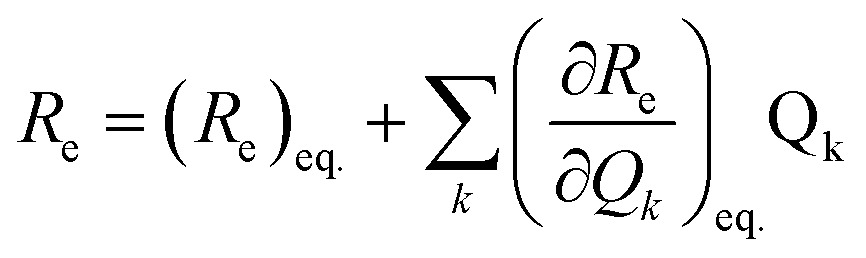



We have recently shown^28^ that the emission spectrum from the smallest ring, ***c*-P6**, can be modeled in terms of HT transitions. As the low emission rate of ***c*-P6** illustrates (Fig. 5b), transition rates are weak for this rigid, highly symmetric molecule suggesting (*R*_e_)_eq._ ≈ 0. Larger porphyrin nanorings, on the other hand, are mechanically more flexible and so can allow direct FC transitions as a result of a static relaxation in symmetry constraints ((*R*_e_)_eq._ > 0). Such effects will allow a recovery of the radiative emission rates towards those of their linear analogues, as shown in Fig. 5b. This interpretation is consistent with our finding of an excitation-energy dependent emission anisotropy for ***c*-P24** (Fig. 4b) and our recent scanning tunneling microscopy (STM) images^25,26,53^ of larger porphyrin rings revealing significant structural deformation. Analysis of STM images allows direct measurement of the deviation *g* from circularity for rings of different sizes.^53^ Here, *g* = *a*/*b* – 1, where *a* and *b* are the longest and shortest distances measureable diametrically across an individual ring examined. Experimentally determined ensemble values of for ***c*-P12** and ***c*-P24** were shown to be compatible with the assumption of a common bending rigidity *κ*_B_ of the butadiyne-linked porphyrin oligomer chain making up the nanorings.^53^ Here we use a simple Monte Carlo simulation in order to predict the dependence of the non-circularity on ring size. Nanorings ***c*-P*n*** are modeled as *n* hard discs linked with flexible bending points, with the thermally equilibrated structure of each ring governed by *κ*_B_ (see ESI† for full details). Fig. 5c shows that the simulated increases appreciably with nanoring size in qualitative agreement with the increase observed in radiative transition rates *k*_r_. While a direct quantitative link between and *k*_r_ is beyond the scope of this study, the correlation gives strong evidence that static distortion leads to the lowest electronic transitions in the nanorings not being fully suppressed. We note that the radiative rate of the largest nanoring (***c*-P24**) approaches that expected for its linear oligomer counterpart (see Fig. 5b). The enhanced flexibility of such large nanorings may also result in significant conformational disorder, which offers an inhomogeneous distribution of sites at which an excitation may eventually localize. For the largest ring, it therefore becomes impossible to distinguish whether its emission becomes dipole-allowed purely as a result of slight distortions away from high symmetry, or because of exciton localization at conformational sub-segments following the initial light absorption. We note that the evolution of the emission spectra with nanorings size supports these arguments. For the smallest ring (***c*-P6**) the emission features a complex vibrational structure, in agreement with strong Herzberg–Teller effects. However, emission from larger rings (*n* = 12) shows spectral features more similar to those of their linear oligomer counterparts, suggesting that non-Condon effects contribute little, in relative terms, to the emission from large nanorings (see ESI†). Our observations have direct analogies with natural chlorophyll ring assemblies such as B850 for which the lowest (*k* = 0) coupled exciton state is known to be dipole forbidden due to symmetry.^18^ However, distortion from circularity and general disorder have been shown to energetically split the higher lying (*k* = ±1) states and transfer oscillator strength to the (*k* = 0) state.^18–20^

### Temperature-dependent photoluminescence

**Fig. 7:**
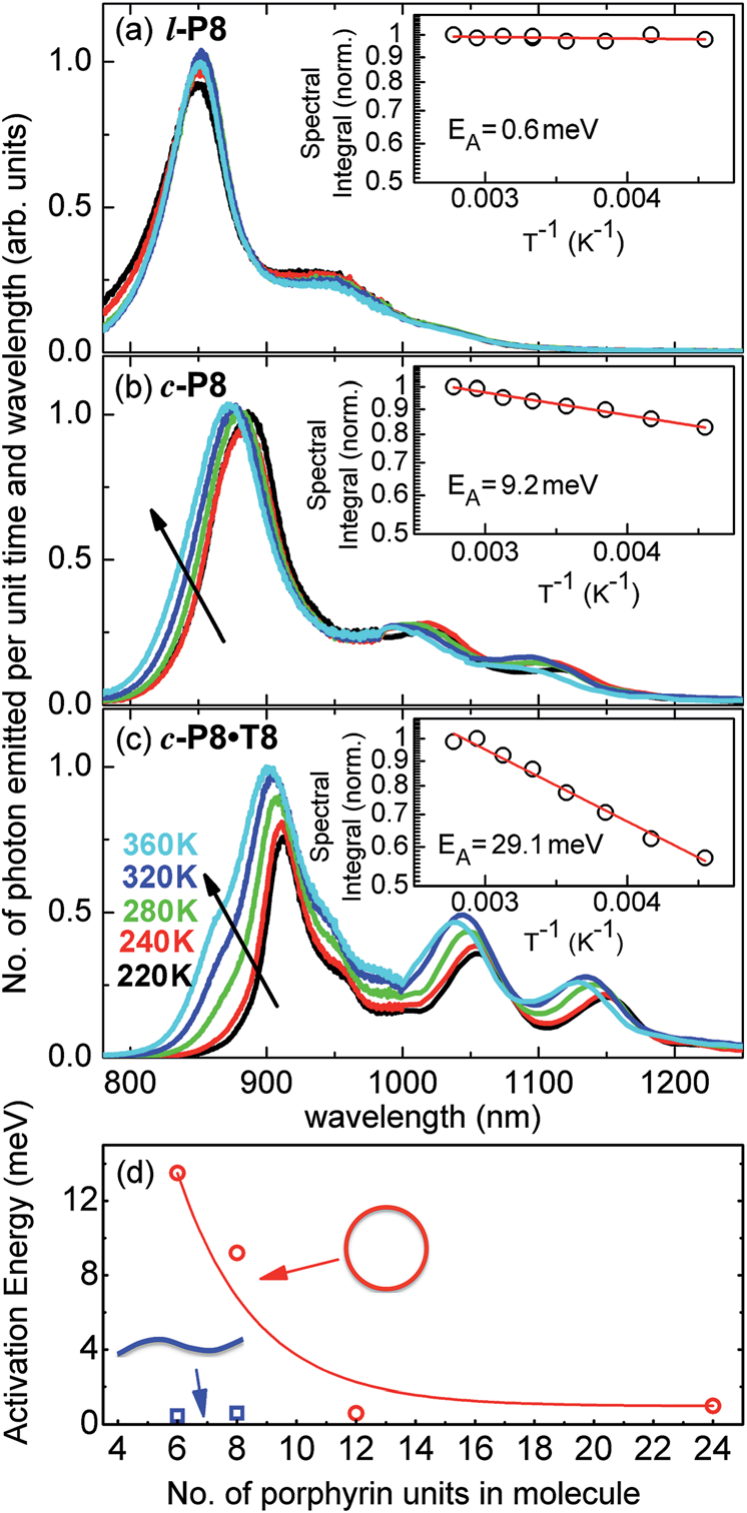
Steady-state PL spectra for (a) ***l*-P8**, (b) ***c*-P8** in toluene/1% pyridine and (c) ***c*-P8·T8** in toluene, at solution temperatures of 220 K (black line), 240 K (red), 260 K (green), 320 K (blue) and 360 K (cyan). The insets show the spectral integral over these spectra (which is proportional to the total number of photons emitted from the sample) as a function of inverse temperature. From these data, the shown activation energy *E*_A_ for thermally enhanced emission was extracted through exponential fitting. (d) Plot of the activation energy, *E*_A_, as a function of the number of porphyrins in the molecule for non-templated, cyclic (open circles) and linear porphyrin oligomers (full squares). Lines are guides to the eye.

The link between disorder and electronic oscillator strength of the lowest state means that natural light harvesting systems would be expected to exhibit thermally activated superradiance.^1^ However, temperature-enhanced luminescence has so far not been observed for natural systems, because the resulting vibrations also electronically decouple the individual chlorophyll chromophores, leading to a breakdown of exciton delocalization.^20,21^ At best, natural light harvesting systems have so far only shown a temperature-independent radiative rate, *e.g.* in the case of the trimeric light-harvesting complex II from spinach^21^ and the LH2 complex from *Rhodobacter sphaeroides*.^20^ For the covalently bonded porphyrin nanorings under investigation here, such thermally activated electronic decoupling should be strongly suppressed. Fig. 7 demonstrates that the 8-porphyrin nanoring ***c*-P8** exhibits pronounced temperature-activated emission enhancement and blue-shifts in accordance with an increase in ring distortion. These changes are entirely caused by an increase in the radiative emission rate with increasing temperature, as the overall PL decay transients (which are dominated by the non-radiative channel) remain unchanged (see ESI†). The insets in Fig. 7 show Arrhenius plots of the spectrally integrated photon emission yield as a function of inverse temperature, from which activation energies for the thermal emission enhancement are extracted through exponential fits. When the ***c*-P8** nanoring is not bound to its **T8** template, the activation energy for emission enhancement is 9.2 meV, while for the more rigid template complex ***c*-P8·T8** this value increases to 29.1 meV. A similar trend is found for ***c*-P6** and ***c*-P6·T6** (see ESI†). These findings support the notion that the thermally enhanced emission rate arises from distortion mechanisms that have higher activation energies for the more rigid template-bound nanorings. Similarly, Fig. 7d shows that the extracted activation energies for template-free rings decrease with increasing ring size, in agreement with the observation that larger rings are more easily distorted. In order to rule out artifacts, we also investigated reference linear oligomers ***l*-P6** and ***l*-P8** (see Fig. 7a and ESI†) for which spectra remain largely unchanged over the same temperature range. We can hence rule out alternative causes for the observed effects, such as changes in the dielectric solvent environment of the nanorings with temperature, which ought to be small in any case, given the low polarity of the toluene solvent. Our simple Monte Carlo simulation predicts a substantial increase in ring non-circularity with increased temperature (see ESI†). Over the lifetime of the excited state, such distortion will appear static and cause a strengthening of the direct FC transition moment (*R*_e_)_eq._ and a blue-shift of the emission, as observed. Dynamic distortions arising from HT coupling, on the other hand, may be augmented by temperature-induced population of higher vibrational levels of the excited-state manifold, as indicated in Fig. 6. Similar HT “hot bands” have been observed, for example, in benzene, where they lead to the appearance of temperature-activated transitions^22,23^ that are shifted by the energy sum of the involved vibrations.^49^ Such temperature-enhanced emission is in contrast with that for natural light-harvesting systems, where the assembly of individual chromophores by scaffold proteins is disrupted with increasing temperature, leading to an electronic decoupling and limiting of wavefunction delocalization.^20,21^

## Conclusion

In summary, we have investigated the nature of the electronic excited states in a family of porphyrin nanorings. We find an ultrafast polarization memory loss for all nanorings ***c*-P6**, ***c*-P8**, ***c*-P12** and ***c*-P24**, with a measured emission anisotropy is *γ* ≤ 0.1 from within 200 fs after excitation. These observations imply that either the absorbing excited state is delocalized over the full nanoring, or that there is extremely fast motion of the electron–hole wavepacket around the nanoring. To elucidate this issue further, we determine the radiative rates, for the nanorings. We find that for the smaller nanorings (*n* = 6–12) rates are suppressed with respect to that for the analogous linear oligomers, but increase gradually with increasing ring size. For these rings, the emission intensity is increasing with temperature, whereas it decreases when the rings are locked into a highly-symmetric geometry with a template.

These findings suggest a complex interplay between electronic wavefunction delocalization, transition rates and symmetry constraints. For small rings, an ultrafast emission depolarization, strongly suppressed radiative rates and spectral features compatible with Herzberg–Teller coupling confirm that both the absorbing and the emitting state must be fully delocalized over the nanoring. For ***c*-P24**, the observed ultrafast emission depolarization shows that the electron–hole wavefunction still samples the full ring within a 200 fs timescale, which is remarkable given its diameter of 10 nm. However, the radiative rate of ***c*-P24** rate becomes identical to that of a linear oligomer. Our experiments and simulations suggest this effect arises *a priori* from the large correlation between radiative rates and the extent to which rings are statically distorted. The reasons for such correlation may be two-fold: on the one hand, static deformations from high symmetry will simply transfer oscillator strength to the lowest excited state. On the other hand, the resulting conformational disorder may offer an inhomogeneous distribution of sites at which an excitation may randomly localize. Our experiments and observations can, at this point, not distinguish between these two scenarios. However, we note that either may be influenced by the increasing conformational flexibility found with increasing nanoring size. For the former scenario, the emitting excited state could still be potentially highly delocalized, while for the latter emission is induced by localization of the exciton along a subsection of the nanoring. Overall, we conclude that for all nanorings investigated, the exciton wave packet is initially highly delocalized along the nanoring. However, while emission from small rings is strongly suppressed by high symmetry, an increase in ring size induces ring deformations that substantially enhance the radiative rate.

Many synthetic porphyrin arrays have previously been studied as LH2 mimics,^14–17,42^ but to the best of our knowledge, the ***c*-P*n*** nanorings are the first such system to show such rapid depolarization (Fig. 4), or thermally activated emission (Fig. 7), or blue-shifted absorption with increasing size (Fig. 3). Ultrafast PL depolarization has previously been observed in other π-conjugated macrocycles based on thiophene^54^ or phenylcarbazole^24^ units, although none of these rings are as large as ***c*-P24** (diameter 10 nm). Furthermore, the previously studied systems do not exhibit indications of temporally sustained delocalization – they do not show the reduced radiative rates or thermally activated superradiance expected for emission from a circularly delocalized state. The photophysical behavior of [*n*]cycloparaphenylenes ([*n*]CPPs) is most similar to that of the ***c*-P*n*** nanorings: they show a PL blue shift with increasing *n*, and smaller [*n*]CPPs with *n* = 6–9 (diameter 0.8–1.2 nm), exhibit low PL quantum yields, which probably result symmetry-reduced *k*_r_, but larger [*n*]CPPs with *n* > 9 are highly emissive and appear to emit from localized states.^38,40,41^

To compare the investigated porphyrin nanorings with natural light-harvesting chlorophyll assemblies in purple bacteria, such as the B850 ring of the LH2 complex, we note that these also exhibit rapid depolarization of the excited state.^6,47^ B850 typically comprises 18 individual bacteriochlorophyll (BChl) units, held in close proximity by the surrounding protein scaffold.^2^ The resulting strong coupling between BChls leads to an excited state that is initially strongly delocalized across many BChls but subsequently suffers disorder-induced localization.^7,8^ The overall process leads to transition anisotropy values of ≤0.1 to be assumed within a timescale of ∼100 fs when B850 is probed in absorption.^47^ However, experimental measurements of fluorescence anisotropy of natural porphyrin ring assemblies have shown maximum initial anisotropy values of 0.4, decaying over sub-picosecond to picosecond timescales indicating rapid exciton hopping,^1,48^ but a PL anisotropy of 0.1 is not reached until almost one picosecond after the initial peak.^48^ Therefore, the synthetic nanorings presented here offer excitonic delocalization around the ring that is at least as fast as that observed in their natural counterparts. In addition, the smaller porphyrin nanorings (***c*-P6** and ***c*-P8**) exhibit thermally enhanced emission, which has so far been elusive for natural systems. The covalent bonding in ***c*-P*n*** nanorings appears to induce a spatial coherence of the excited state that is remarkably robust against disorder-induced weakening of the electronic coupling between porphyrin units. These results suggest a host of technological applications for light-harvesting materials that match the efficiency of their natural counterparts, and allow for an exploration of the fundamental science of large molecular macrocycles supporting highly delocalized electronic states.
