# Supplementary material for: Ultrafast delocalization of excitation in synthetic light-harvesting nanorings
Source: Chem Sci. 2014 Sep 16;6(1):181–9. doi: 10.1039/c4sc02424a (PMC5424671; doi:10.1039/c4sc02424a)
Supplement: Supplementary file 1 [file SC-006-C4SC02424A-s001.pdf]

Electronic Supplementary Material (ESI) for Chemical Science.
This journal is © The Royal Society of Chemistry 2014

Electronic Supplementary Information

Ultrafast Delocalization of Excitation in Synthetic Light-Harvesting Nanorings

Chaw-Keong Yong,[†] Patrick Parkinson,[†] Dmitry V. Kondratuk,[‡] Wei-Hsin Chen,[†] Andrew Stannard,[¶] Alex Summerfield,[¶] Johannes K. Sprafke,[‡] Melanie C. O'Sullivan,[‡] Peter H. Beton,[¶] Harry L. Anderson[‡] and Laura M. Herz*[†]*

[†] *University of Oxford, Department of Physics, Clarendon Laboratory, Parks Road, Oxford, OX1 3PU, U.K.*
[‡] *University of Oxford, Department of Chemistry, Chemistry Research Laboratory, Oxford, OX1 3TA, U.K.*
[¶] *School of Physics & Astronomy, University of Nottingham, Nottingham, NG7 2RD, U.K.*

Email: l.herz@physics.ox.ac.uk & harry.anderson@chem.ox.ac.uk

Chemical Synthesis and Characterization of Porphyrin Molecules.

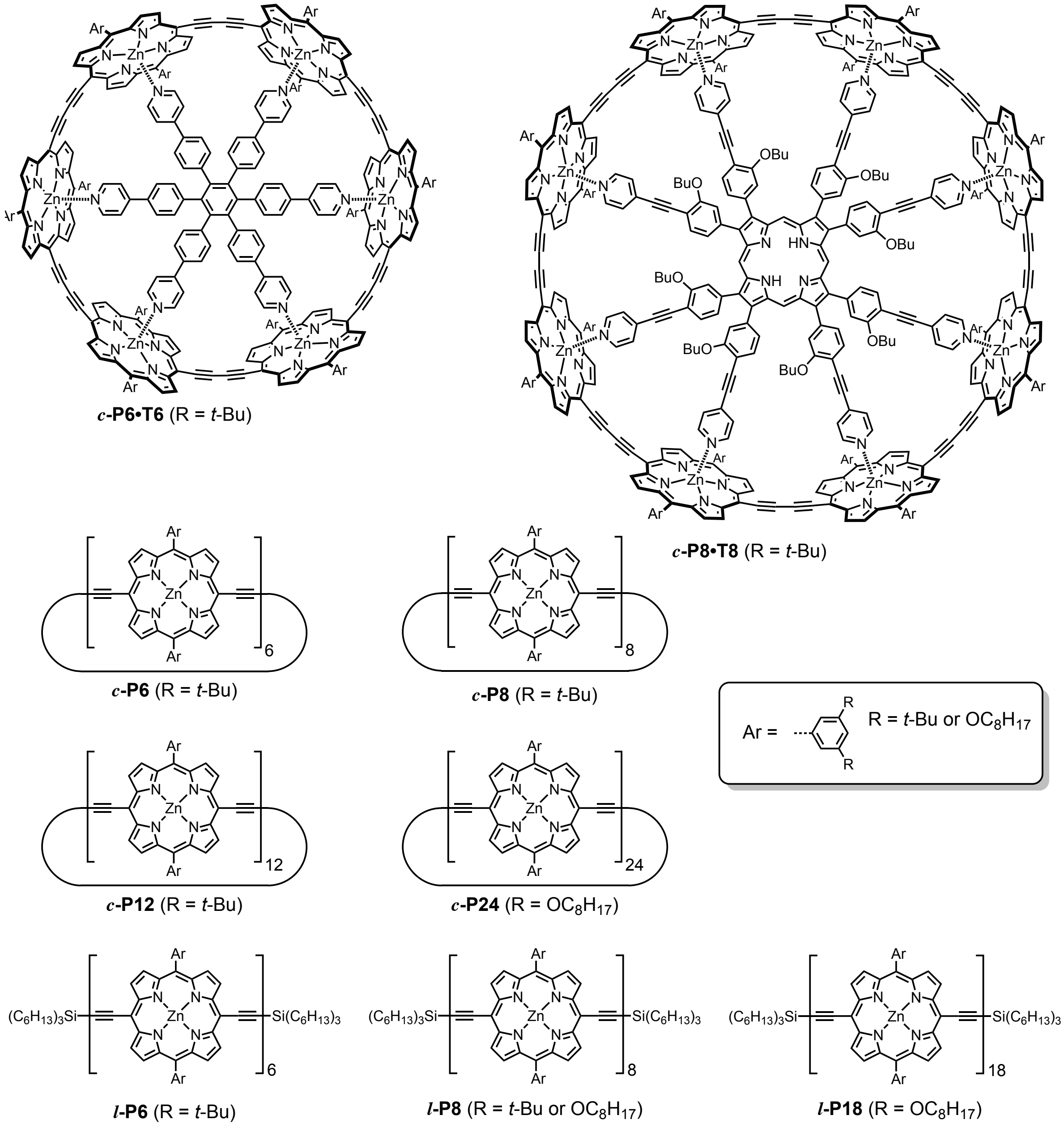

Figure 1: Detailed chemical structures of the porphyrin oligomers used in this study. The sidechain (R) has no significant effect on the absorption or photoluminescence spectra of the compounds, but it influences the solubility, aggregation behaviour and ease of purification. Versions of the oligomers with R = t-Bu or R = OC_8H_{17} were used, as indicated above. In the case of the linear octamer ***l*-P8**, material with R = t-Bu was used for photoluminescence anisotropy experiments whereas octamer with R = OC_8H_{17} was used in temperature-dependent photoluminescence experiments.

All photoluminescence experiments were carried out using solutions of zinc porphyrin oligomers in toluene containing 1% by volume of pyridine, except in the cases of ***c*-P6·T6** and ***c*-P8·T8** which were studied in pure toluene. The pyridine ligand coordinates to the zinc centres of the porphyrin units, increasing the solubility and preventing aggregation. The chemical structures of the zinc porphyrin oligomers used in this study are shown in Figure 1. These materials were synthesised and characterised using previously published procedures.[1–9] All samples were rigorously purified by gel permeation chromatography (GPC), and their purity was checked by 500 MHz ^{1}H NMR, MALDI-TOF mass spectrometry and analytical GPC. The cyclic hexamer and its template complex, ***c*-P6** and ***c*-P6·T6**, were prepared directly from the porphyrin monomer ***l*-P1** as described in Ref. 4. The cyclic octamer and its template complex, ***c*-P8** and ***c*-P8·T8**, were prepared from ***l*-P2** using the **T8** template, using the method described in Ref. 3.

Monodisperse linear oligomers ***l*-P6** and ***l*-P8** were synthesised by coupling mono-silylated precursors as described in references 2,6–9. The synthesis and characterisation of ***l*-P18** is described as follows; ***l*-P10** emerged as a by-product of the process but was not used in the study reported here.

THS-protected linear porphyrin 8-mer ***l*-P8**$_{\mathrm{THS,THS}}$ (54.0 mg, 5.8 μmol) was dissolved in CH_2Cl_2 (30.0 mL) and the solution saturated with N_2. Tetra-*n*-butylammonium fluoride (1.00 M in THF, 160 μL, 0.160 mmol) was added and the reaction mixture stirred at 20°C for 1 h under N_2 atmosphere. The reaction was quenched with MeOH (1.0 mL), the volume reduced, the ***l*-P8**$_{\mathrm{H,H}}$ precipitated with MeOH (50 mL) was filtered off and dried in high vacuum. All obtained ***l*-P8**$_{\mathrm{H,H}}$ and half-deprotected porphyrin monomer ***l*-P1**$_{\mathrm{H,THS}}$ (308 mg, 0.225 mmol) were dissolved in a mixture of toluene (100 mL) and pyridine (1.0 mL). A catalyst solution was prepared by dissolving dichlorobis(triphenylphosphine)-palladium(II) (107 mg, 0.152 mmol), copper(I) iodide (263.3 mg, 1.38 mmol) and 1,4-benzoquinone (248.8 mg, 2.30 mmol) in a mixture of toluene (80.0 mL) and freshly distilled *i*-Pr_2NH (18.0 mL) and added to the solution of ***l*-P8**$_{\mathrm{H,H}}$ and ***l*-P1**$_{\mathrm{H,THS}}$. The reaction mixture was stirred at 20°C for 3 h, after which half of the initial amount of the catalyst solution was added again and the mixture stirred at 60°C for 2 h. Once UV-vis spectroscopy showed

no changes, the reaction mixture was passed through a short silica column ($CHCl_3$/1% pyridine) to remove the catalysts and then over a size exclusion column (Biobeads SX-1 in toluene/1% pyridine) to remove the 1,4-benzoquinone. Preparative GPC (toluene/10% pyridine) followed by recycling GPC yielded 242 mg (79 %) of ***l*-P2**$_{THS,THS}$, 47 mg (70 %) of ***l*-P10**$_{THS,THS}$ and 4 mg (7 %) of ***l*-P18**$_{THS,THS}$ as brown solids.

***l*-P10**$_{THS,THS}$: ^{1}H-NMR (400 MHz, $CDCl_3$/1% d_5-pyridine): δ_H 9.77–9.73 (m, 36H, -ArH$_\beta$), 9.54 (d, 4H, J = 4.5 Hz, -ArH$_\beta$), 8.96–8.93 (m, 36H, -ArH$_\beta$), 8.84 (d, 4H, J = 4.4 Hz, -ArH$_\beta$), 7.28–7.21 (m, 40H, Ar-H$_{ortho}$), 6.80–6.77 (m, 20H, Ar-H$_{para}$), 3.99 (m, 80H, -OCH_2), 1.76–1.67 (m, 92H, -CH_2), 1.65–1.59 (m, 12H, -CH_2), 1.42–1.07 (m, 424H, -CH_2), 0.89–0.85 (m, 12H, -CH_2), 0.74–0.66 (m, 138H, -CH_3); *m/z* (MALDI-ToF) 11419 ($C_{716}H_{898}N_{40}O_{40}Zn_{10}$, M^+ requires 11415).

***l*-P18**$_{THS,THS}$: ^{1}H-NMR (400 MHz, $CDCl_3$/1% d_5-pyridine): δ_H 9.74–9.70 (m, 68H, -ArH$_\beta$), 9.51(d, 4H, J = 4.5 Hz, -ArH$_\beta$), 8.93–8.89 (m, 68H, -ArH$_\beta$), 8.81 (d, 4H, J = 4.5 Hz, -ArH$_\beta$), 7.23–7.17 (m, 72H, Ar-H$_{ortho}$), 6.77–6.75 (m, 36H, Ar-H$_{para}$), 3.94 (m, 144H, -OCH_2), 1.71–1.64 (m, 156H, -CH_2), 1.61–1.55 (m, 12H, -CH_2), 1.35–0.99 (m, 774H, -CH_2), 0.85–0.80 (m, 12H, -CH_2), 0.70–0.60 (m, 234H, -CH_3); *m/z* (MALDI-ToF) 20181 ($C_{1260}H_{1554}N_{72}O_{72}Zn_{18}$, M^+ requires 20093).

^{1}H NMR, MALDI-ToF and GPC characterisation data of ***l*-P10**$_{THS,THS}$ and ***l*-P18**$_{THS,THS}$ are shown in Figures 2–6.

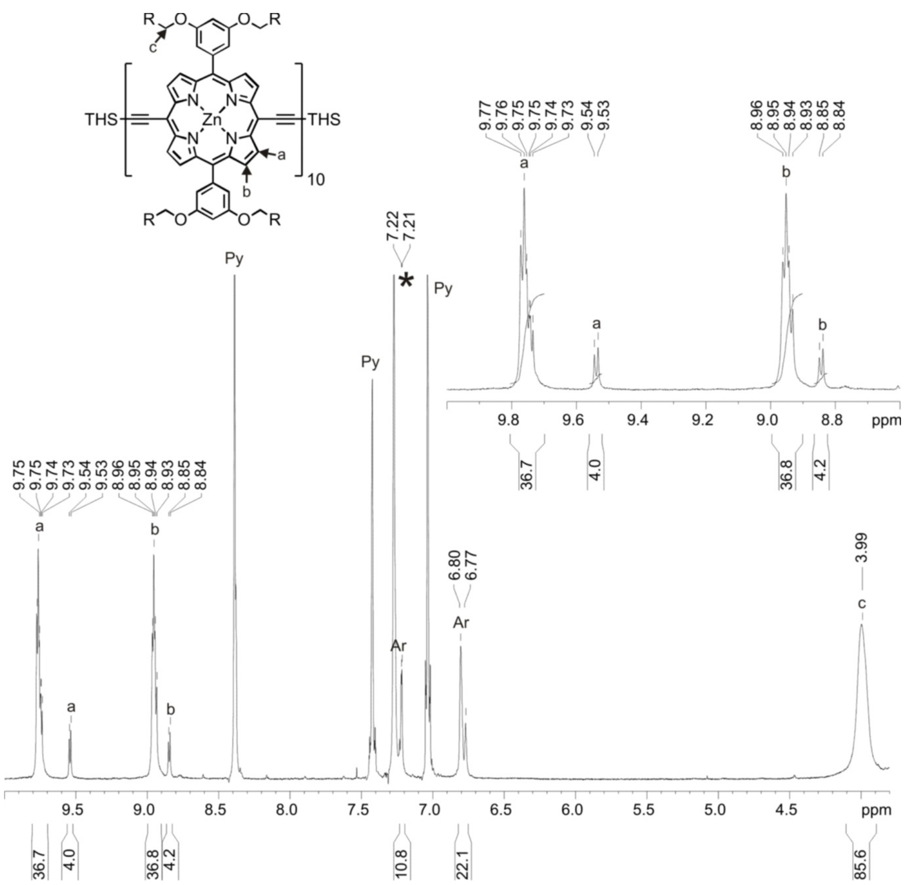

Figure 2: ^{1}H NMR spectrum of ***l*-P10**$_{THS,THS}$ with zoom on the β-pyrrole region (400 MHz, $CDCl_3$/1% d_5-pyridine).

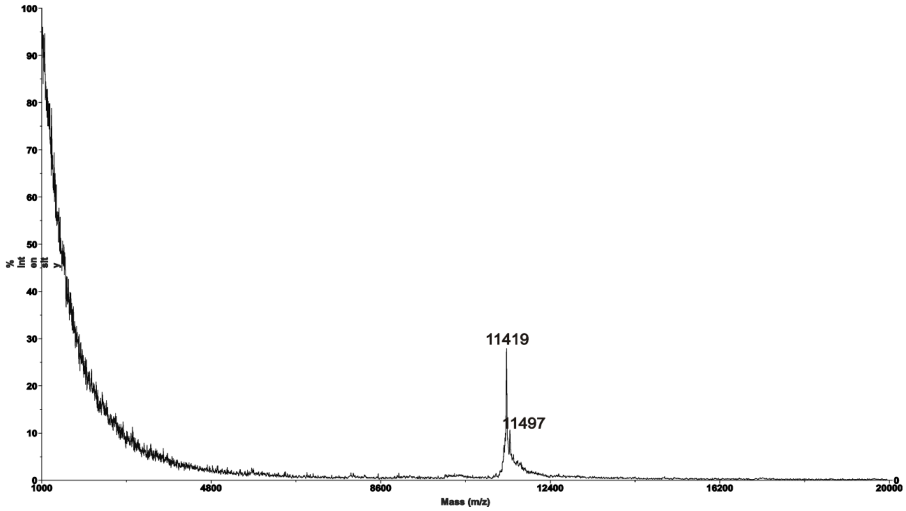

Figure 3: MALDI-ToF analysis of ***l*-P10**$_{THS,THS}$. The major peak corresponds to ***l*-P10**$_{THS,THS}$ (*m/z* 11419, expected 11415). DCTB was used as matrix.

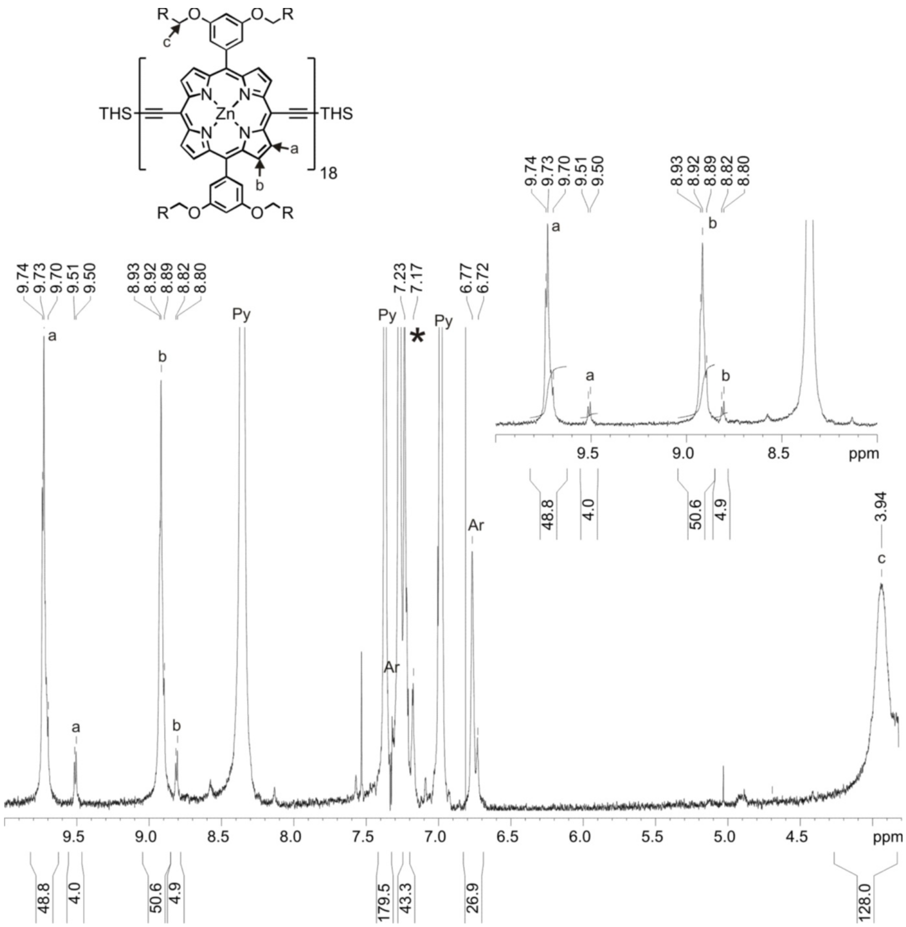

Figure 4: ^{1}H NMR spectrum of ***l*-P18**$_{\mathrm{THS,THS}}$ with zoom on the β-pyrrole region (400 MHz, $CDCl_3$/1% d_5-pyridine).

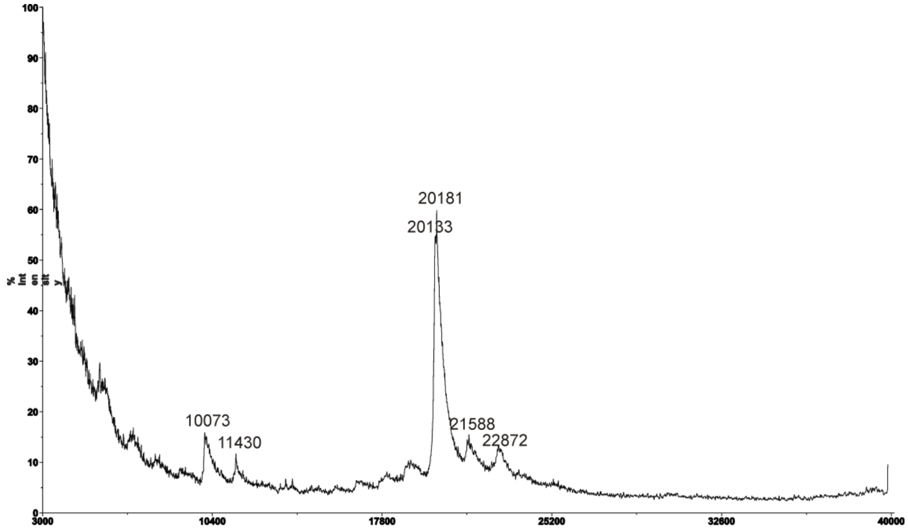

Figure 5: MALDI-ToF analysis of ***l*-P18**$_{\mathrm{THS,THS}}$. The major peak corresponds to ***l*-P18**$_{\mathrm{THS,THS}}$ (*m/z* 20181, expected 20093). DCTB was used as matrix.

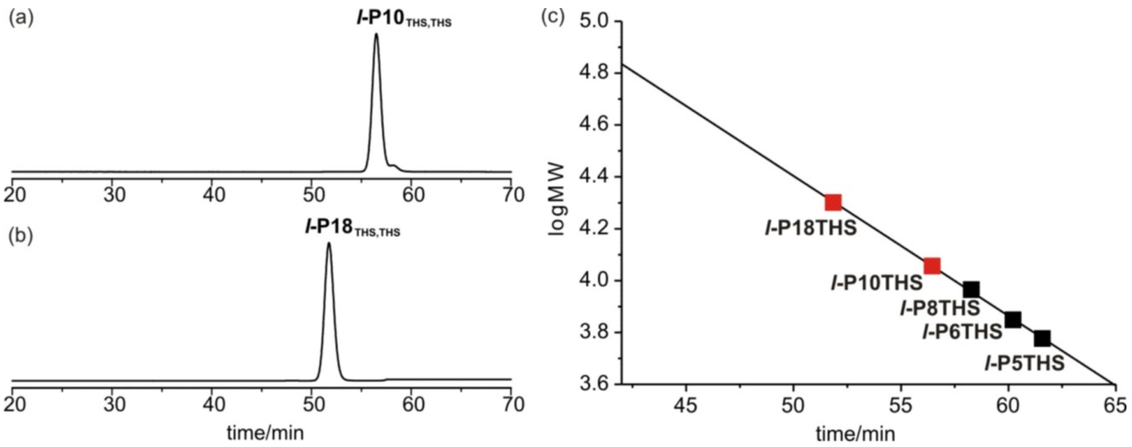

Figure 6: Analytical GPC traces of (a) ***l*-P10**$_{THS,THS}$ and (b) ***l*-P18**$_{THS,THS}$ (toluene/1% pyridine, detection at 500 nm). (c) Calibration of the used GPC columns with previously reported linear porphyrin oligomers (black squares) plotted together with the corresponding values for ***l*-P10**$_{THS,THS}$ and ***l*-P18**$_{THS,THS}$ (red squares).

Steady-state Absorption and Emission Spectra

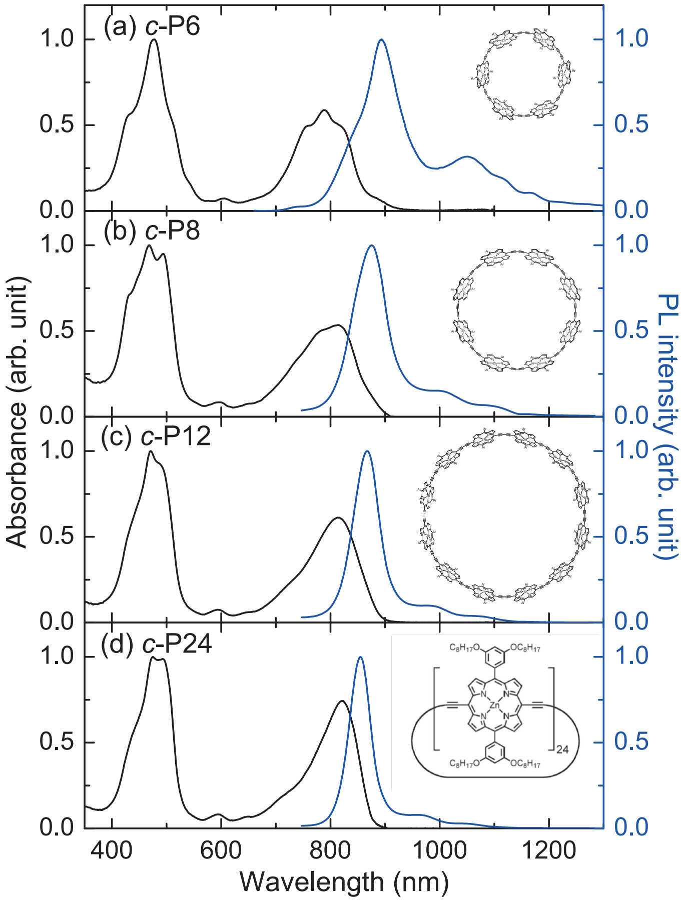

Figure 7: Normalized steady-states absorption (black lines) and photoluminescence (blue lines) spectra at 295 K of cyclic porphyrins (a) *c*-P6, (b) *c*-P8, (c) *c*-P12 and (d) *c*-P24 in toluene/1% pyridine. The emission was recorded after excitation at 770 nm (1.61 eV).

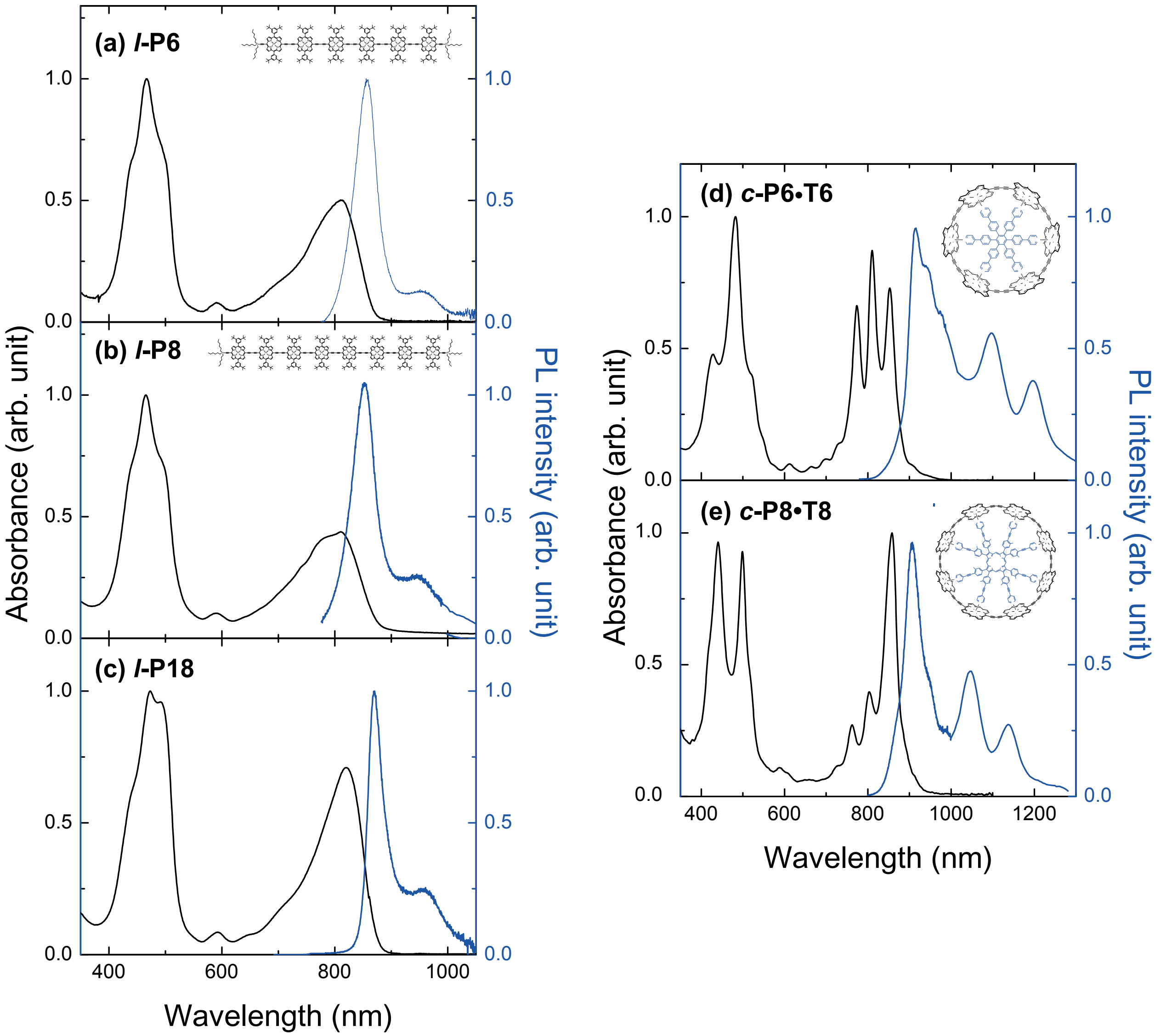

Figure 8: Normalized steady-state absorption (black lines) and photoluminescence (blue lines) spectra at 295 K for (a) *l*-P6, (b) *l*-P8, (c) *l*-P18, in toluene/ 1% pyridine, and (d) *c*-P6·T6 and (e) *c*-P8·T8 in toluene. The emission was recorded after excitation at 770 nm (1.61 eV).

PL Anisotropy Dynamics

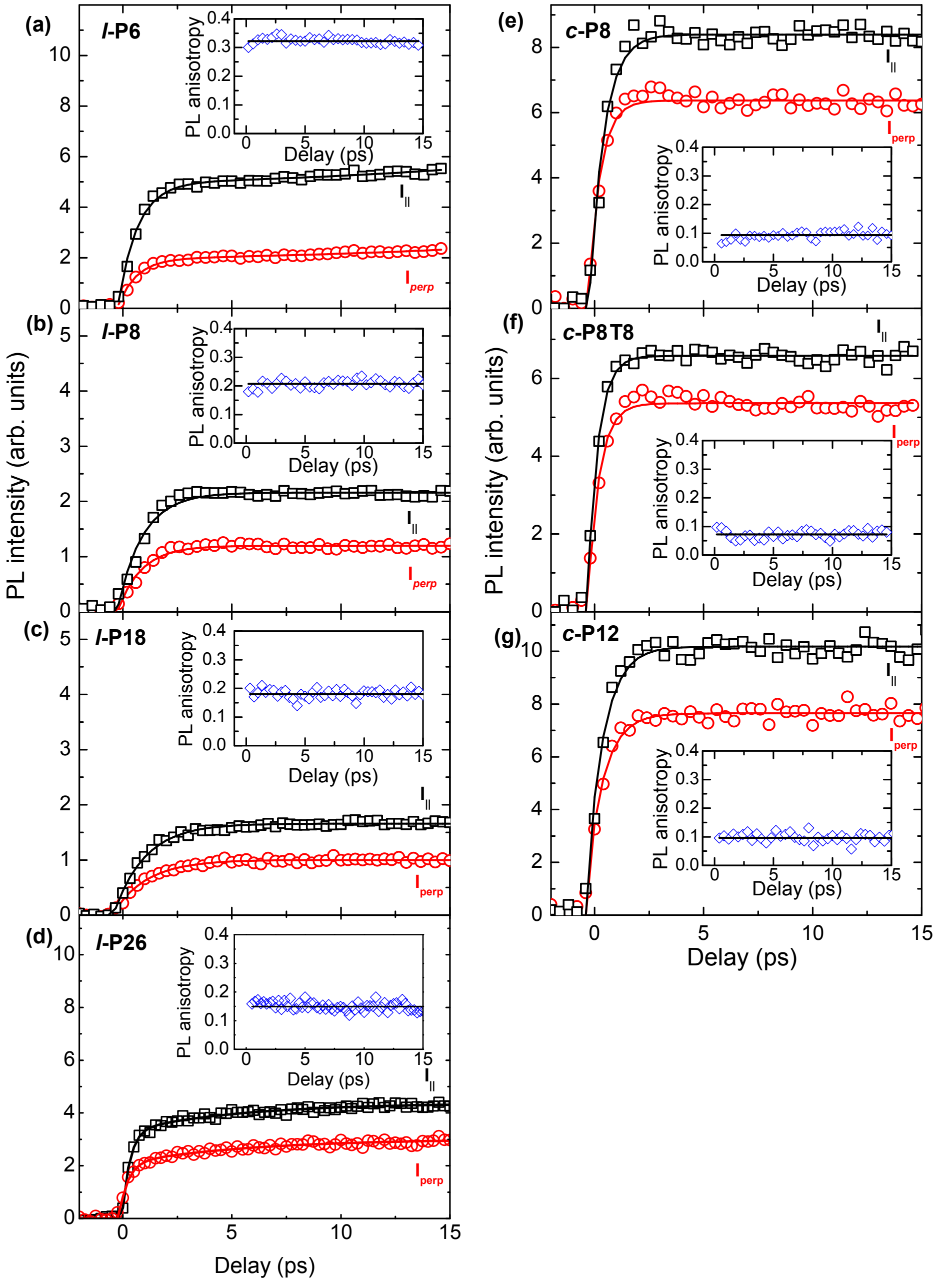

Figure 9: PL transients of (a) *l*-P6, (b) *l*-P8, (c) *l*-P18, (d) *l*-P26, (e) *c*-P8, and (g) *c*-P12 in toluene/1% pyridine, and (f) *c*-P8·T8 in toluene, after excitation at 770 nm and detection at the emission wavelength corresponding to the peak emission of their steady-state PL spectra (*l*-P6: 876 nm, *l*-P8: 856 nm, *l*-P18: 869 nm, *l*-P26: 870 nm, *c*-P8: 876 nm, *c*-P8·T8: 930 nm, *c*-P12: 866 nm). The samples were excited with the ultrashort pulse polarization parallel ($I_{\parallel}$, black squares) and perpendicular (I_{perp}, red circles) to the PL detection polarization. Inset: The derived PL polarization anisotropy γ as a function of time. The solid lines are guides to the eyes.

Figure 9 shows the time evolution of the photoluminescence intensity components with polarization parallel ($I_{\|}$) and perpendicular ($I_{\perp}$) to the excitation polarization, for four linear porphyrin oligomers, *l*-P6, *l*-P8, *l*-P18 and *l*-P26, and three porphyrin nanorings, *c*-P8, *c*-P8·T8 and *c*-P12. The insets show the derived PL polarization anisotropy $\gamma = (I_{\|} - I_{\perp})/(I_{\|} + 2I_{\perp})$. For the nanorings, values of γ are generally near 0.1, the theoretically expected value for polarization memory loss in a two-dimensional plane of the molecule. For the linear oligomers, an initial PL polarization anisotropy of 0.32, 0.21, 0.18 and 0.15 is observed for *l*-P6, *l*-P8, *l*-P18 and *l*-P26, respectively, where the former two are in agreement with a previous study on identical molecules which also determined an initial anisotropy of 0.36 for the linear tetramer (*l*-P4) of the same series.[10] This study demonstrated that only for short oligomers a PL anisotropy close to 0.4 was found, the theoretically expected value for a randomly oriented distribution of non-interacting linear dipoles.[11] For longer oligomers, the early-time anisotropy (following light absorption and nuclear relaxation) was observed to be smaller, the longer the porphyrin oligomer.[10] These effects were shown to be related to the "worm-like" nature of longer molecules which may be substantially bent, and hence an ultrafast reorientation of the oscillating dipole moment may originate from excitation self-trapping following fast vibrational relaxation of the molecule. The PL anisotropy dynamics for the "linear" porphyrin molecules can hence be understood in terms of dynamic exciton self-localization that leads to an ultrafast re-orientation of the emitting dipole. Such depolarization effects will be more pronounced the more curved the conjugated segments are that sustain the excitation, as described previously.[10]

PL Intensity Transients

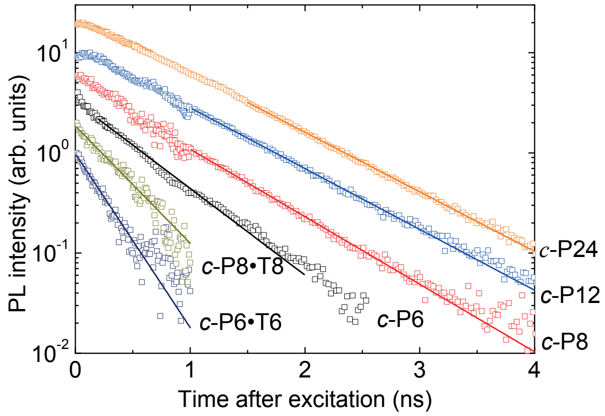

Figure 10: PL transients of templated porphyrin nanorings *c*-P6·T6, *c*-P8·T8 in toluene, and free nanorings *c*-P6, *c*-P8, *c*-P12 and *c*-P24 in toluene/1% pyridine detected at the emission wavelength corresponding to the peak emission of their steady-state PL. The solid lines are fits of a single-exponential function to the data, from which the total decay rate was extracted.

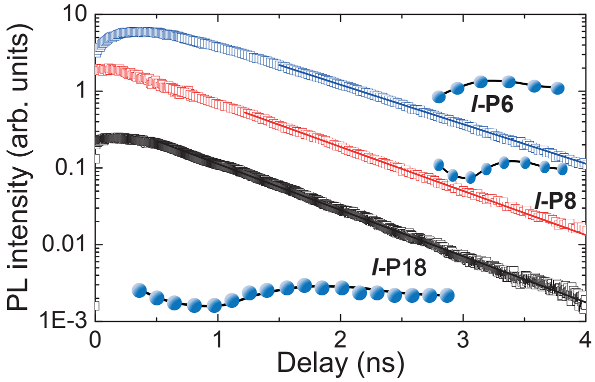

Figure 11: PL transients of *l*-P6, *l*-P8 and *l*-P18, in toluene/ 1% pyridine detected at the emission wavelength corresponding to the peak emission of their steady-state PL (*l*-P6: 876 nm, *l*-P8: 856 nm, *l*-P18: 869 nm). The solid lines are fits of a single-exponential function to the data, from which the total decay rate was extracted.

Time-dependent PL intensity decay transients were determined from the two measured intensity components ($I_{\|}$, with polarization parallel, and $I_{\perp}$ with polarization perpendicular to the excitation polarization) as $I = I_{\|} + 2I_{\perp}$ and are plotted in Fig. 10 for cyclic and Fig. 11 for linear porphyrins. From monoexponential fits to these data ($I = I_0 \exp(-k_{total}t)$) the overall decay rate k_{total} was determined, which is composed of both radiative (k_r) and non-radiative (k_{nr}) parts.

Quantum Yield

Calculation of the fluorescence quantum yield of porphyrin compounds is complicated by the lack of well-characterized quantum yield standards in the near-infrared region. Accordingly, two mutually complementary techniques were used to ensure an accurate determination of the quantum yield of one compound (*l*-P6) to be used as a reference material. Accordingly, the yield of *l*-P6 was determined using both:

A) An Integrating Sphere Approach. An absolute measurement of quantum yield may be made using a broadband integrating sphere and spectrally corrected spectrometer, following an approach as described by de Mello *et al.*[12] Briefly: three spectral measurements are made, one without a sample present (referred to as I_{las}), one with the sample in the sphere and directly in the laser beam (I_{in}) and one with the sample in the sphere but removed from the direct beam and illuminated by scattered laser light only (I_{out}). The concentration of the sample is carefully controlled to have less than ∼10% absorption at any wavelength to avoid self-absorption effects. The single-pass absorption of the sample can be calculated by

$$A = 1 - \frac{I_{in}(\lambda_{laser})}{I_{out}(\lambda_{laser})}, \qquad (1)$$

where λ_{laser} indicates the number of photons in the spectral region of the exciting laser. From this, the quantum yield may be calculated using

$$QY = I_{in}(\lambda_{PL}) - (1 - A)\frac{I_{out}(\lambda_{PL})}{I_{las}(\lambda_{laser})A}, \qquad (2)$$

where λ_{PL} is the number of photons in the spectral region of the photoluminescence. Under excitation into the Soret band using a 450 nm LED source, a quantum yield of 0.263 was calculated, while excitation into the Q_X band using a 770 nm laser diode resulted in a quantum yield of 0.256. This technique can provide high systematic accuracy, but – due to the direct comparision of laser and photoluminescence on a single scan – can be challenging for low quantum yield samples.

B) A Relative Approach. A relative measurement of quantum yield can be made by comparison of the absorption and emission of a sample in comparison to that of a reference standard, using the relationship

$$QY(\text{Sample}) = \frac{\text{I}_{\text{Standard}}(\lambda_{\text{PL}})}{\text{I}_{\text{Sample}}(\lambda_{\text{PL}})} \times \frac{\text{ABS}_{\text{Sample}}(\lambda_{\text{laser}})}{\text{ABS}_{\text{Standard}}(\lambda_{\text{laser}})} \times \text{QY}(\text{Standard}). \tag{3}$$

From the integrated area of the emission spectra ($I(\lambda_{\text{PL}})$) and the absorbance at the excitation wavelength ($ABS(\lambda_{\text{laser}})$) the quantum yield of sample may be determined. Two dilute fluorescence standards were used; Rhodamine-6G in ethanol for excitation at 475 nm (QY=0.95[13]), and IR140 in ethanol for excitation at 740 nm (QY=0.167[14]). Low sample concentrations were used (less than 10% absoption across the spectrum), along with low excitation powers of 5 mW (as provided by an pulsed Ti:Sapphire oscillator). For excitation into the Soret band (with Rhodamine-6G reference) a quantum yield of 0.316 was measured for *l*-P6, while excitation into the Q_X band (with IR140 reference) provided a value of 0.279. This technique has high precision, but can affected by non-ideality in the reference samples.[14]

As a representative value of the quantum yield of the *l*-P6 sample, the algebraic mean value of 0.28±0.04 was chosen. This value is comparable to that measured for other Zn-porphyrin oligomers emitting in the near infra red.[15] Using the 'Relative Approach' as described above, all samples were characterized with references to the *l*-P6. The resulting values for the quantum yield are displayed in Table 1 for the investigated range of cyclic and linear porphyrin compounds.

Table 1: Quantum Yield of Porphyrins

Material	Quantum Yield
l-P6 (reference)	0.28
l-P8	0.34
l-P18	0.28
c-P6	0.015
c-P6·T6	0.0042
c-P8	0.063
c-P8·T8	0.025
c-P12	0.16
c-P24	0.26

Radiative and Non-radiative Decay Rates

Using the extracted Quantum Yield QY, the overall decay rate k_{total} extracted from the time-dependent PL intensity decay was separated into its radiative (k_r) and non-radiative (k_{nr}) parts through

$$k_r = QY \times k_{total} \text{ and } k_{total} = k_r + k_{nr}.$$

The resulting non-radiative and radiative decay rates are shown in Figure 12 for cyclic and linear porphyrins as a function of the number of porphyrin monomers incorporated in the molecule. For all compounds, the non-radiative decay rates is at a factor of two larger than the radiative rate. The observed PL transients are hence dominated by the non-radiative contribution to the overall decay.

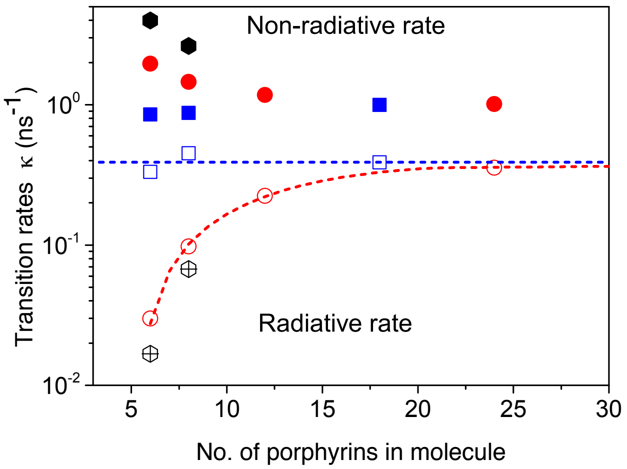

Figure 12: Radiative (open symbols) and non-radiative (full symbols) transition rates as a function of the number of porphyrins in the molecule in solution. The transition rates for free porphyrin nanorings, templated porphyrin nanorings and porphyrin linear oligomers are represented by the red circles, black hexagons and blue squares, respectively. All lines are guides to the eye.

Temperature Dependent Time-Integrated PL

Figure 13 and Figure 14 display the time-integrated PL emission spectra for *c*-P6, *c*-P6·T6, *c*-P12, *c*-P24 and *l*-P6 as a function of solution temperature between 220 K and 360 K. The inset shows the respective integral over these spectra (which are proportional to the total number of photons emitted across the spectrum) as a function of inverse absolute temperature. Fitting these data with an Arrhenius function allows the extraction of the shown activation energies E_A for temperature-activated photon emission rates. For *c*-P6 and *c*-P6·T6 (as well as *c*-P8 and *c*-P8·T8 - see main manuscript) sizeable activation energies are observed, while for the larger nanorings and for the linear molecules, E_A is comparatively small. For the 6-nanorings, templating leads to an increase in activation energy, similar to observations made for the 8-nanorings (see main manuscript). Note however, that for the 6-rings, this increase is smaller, which we attribute to the presence of a peak (at 920 nm) in the emission of *c*-P6·T6 that shows opposite trends to the rest of the spectrum (i.e. an decrease in PL efficiency with increasing temperature). This peak has been identified in a previous study[4] as arising from an impurity emission. Hence the correct activation energy for an impurity-free solution of *c*-P6·T6 is likely to be larger than the stated value of $E_A = 20.5$ meV.

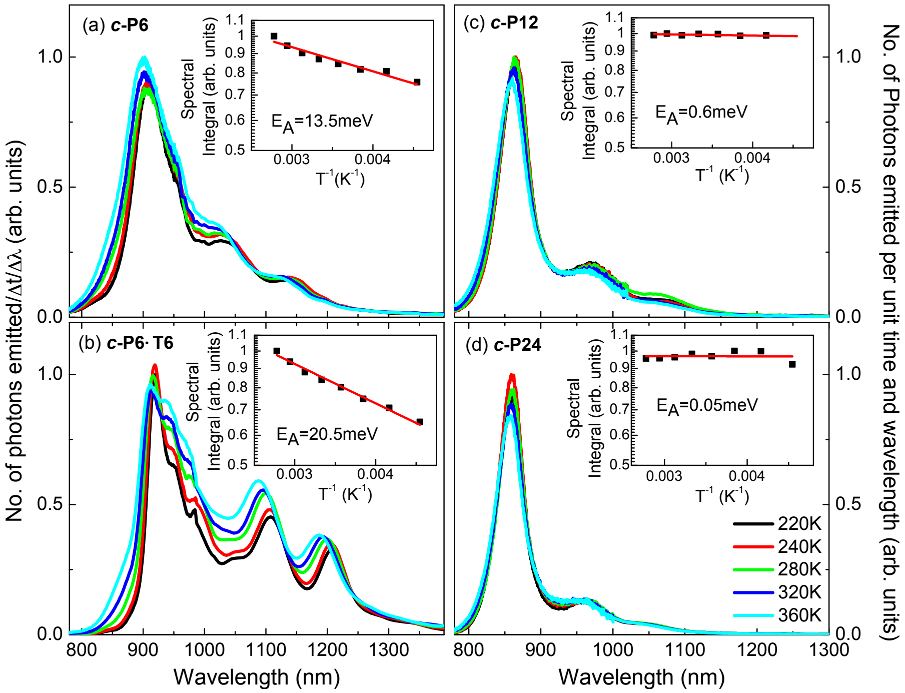

Figure 13: Steady-state photon emission intensity spectra for (a) *c*-P6 in toluene/ 1% pyridine, (b) *c*-P6·T6 in toluene, (c) *c*-P12 in toluene/ 1% pyridine, and (d) *c*-P24 in toluene/ 1% pyridine at sample temperatures of 220 K (black line), 240 K (red), 260 K (green), 320 K (blue) and 360 K (cyan). The insets show the spectral integral over these spectra (which is proportional to the total number of photons emitted from the sample) as a function of inverse temperature. From these data, the shown activation energy E_A for thermally enhanced emission was extracted through exponential fitting.

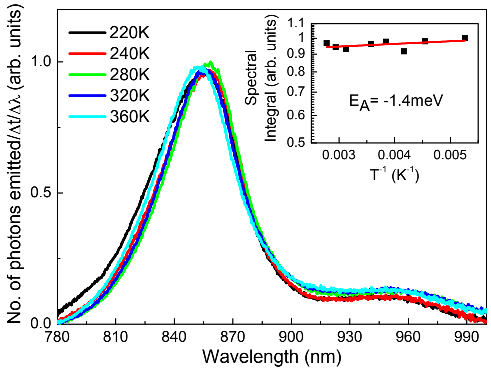

Figure 14: Steady-state photon emission intensity spectra for *l*-P6 in toluene/1% pyridine at sample temperatures of 220 K (black line), 240 K (red), 260 K (green), 320 K (blue) and 360 K (cyan). The insets show the spectral integral over these spectra (which is proportional to the total number of photons emitted from the sample) as a function of inverse temperature. From these data, the shown activation energy E_A for thermally enhanced emission was extracted through exponential fitting.

PL decay dynamics at different solution temperatures

Figure 15 displays the PL emission decay curves for *l*-P8, *c*-P8 and *c*-P8·T8 as a function of solution temperature. For the cyclic molecules, no change in decay dynamics is observed. As shown above, the PL decay for cyclic compounds is dominated significantly by non-radiative decay channels and hence the observed increase in PL emission intensity with increasing temperature can only be caused by an increase in the radiative emission rate.

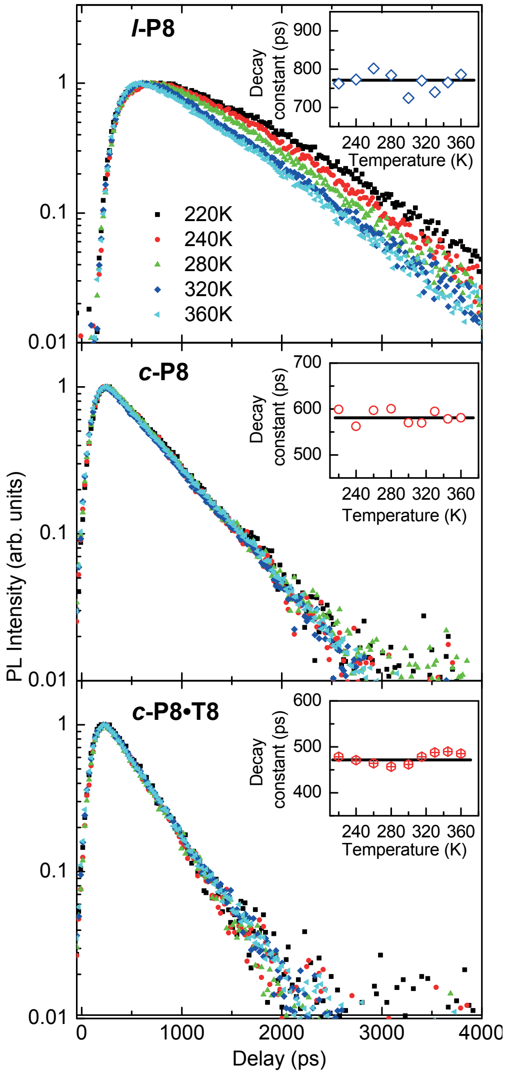

Figure 15: PL decay transients for (a) *l*-P8, (b) *c*-P8 in toluene/1% pyridine and (c) *c*-P8·T8 in toluene taken with the TCSPC technique (time resolution of 180 ps) at different solution temperatures between 220 K and 360 K. Insets show the decay time constant (extracted from mono-exponential fits to the decay tails) as a function of temperature. The solid lines are guides to the eye.

PL energy shifts with solution temperature

Figure 16 shows the average energy of photons emitted from linear porphyrin oligomers and un-templated porphyrin nanorings as a function of inverse temperature. Average energy values $\langle E \rangle$ were calculated through numerical integration using

$$\langle E \rangle = \frac{\int N(E) E dE}{\int N(E) dE} \tag{4}$$

where E is the photon energy and $N(E)dE$ the number of photons emitted within the interval dE of the spectrum. For linear oligomers no energetic shifts are discernable in analogy with the absence of temperature-activated emission enhancement (see main text). For porphyrin nanorings, energetic shifts are visible, which are larger, the smaller the diameter of the nanoring. These observations are in agreement with temperature activated higher-energy emission resulting e.g. from excitations residing on statically more distorted rings, or transitions involving the absorption of a vibration with correct symmetry.

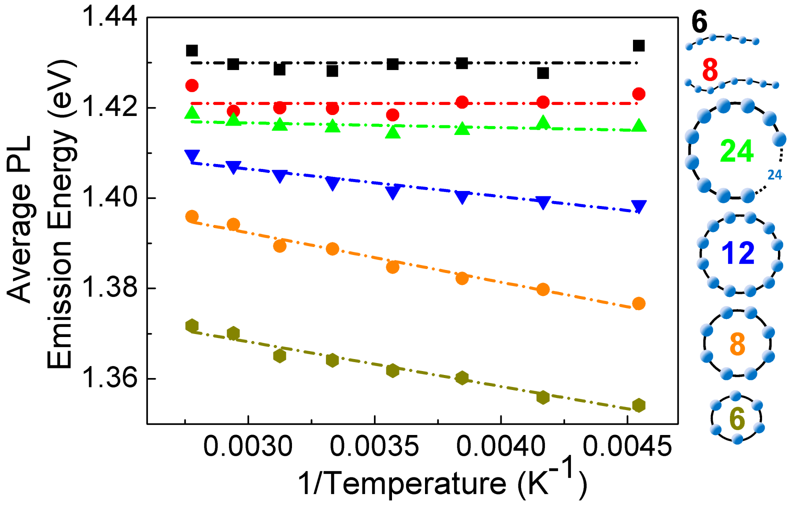

Figure 16: Average energy of photons emitted from porphyrin nanorings and porphyrin oligomers plotted as a function of inverse temperature for (from top to bottom) *l*-P6, *l*-P8, *c*-P24, *c*-P12, *c*-P8 and *c*-P6 in toluene/1% pyridine. Lines are guides to the eye.

Monte Carlo Simulation of Porphyrin Nanoring Conformations

A Monte Carlo model was used to generate thermally-equilibrated nanoring configurations to simulate the average non-circularity $\overline{g}$ in an ensemble of nanorings containing n porphyrins.[16]
The expression for the bending energy of a nanoring is a discretised analogue of the elastic bending energy of a continuous closed loop,

$$U_B = \frac{\kappa_B}{2} \oint C^2(s)\,\mathrm{d}s,$$

where κ_B is the bending rigidity, C is the local curvature, and s is the loop coordinate. The model of a c-Pn nanoring has n bending points, labelled by index i, defined at positions $\{\mathbf{r}_i\}$. Bending points are connected via vectors $\{\mathbf{s}_i\}$, where $\mathbf{s}_i = \mathbf{r}_i - \mathbf{r}_{i-1}$ (periodic boundaries apply). The total energy of a nanoring is the sum of bending terms at positions $\{\mathbf{r}_i\}$ and stretching terms of vectors $\{\mathbf{s}_i\}$,

$$U = \frac{\kappa_B}{2}\sum_i^n C_i^2 \Delta s_i + \frac{\kappa_S}{2}\sum_i^n \left(|\mathbf{s}_i| - L\right)^2,$$

where C_i and Δs_i are local curvature and segment length at the ith bending point, κ_s is stretching stiffness, and L is equilibrium separation between bending points. Local curvatures and segment lengths may be expressed in terms of vectors $\{\mathbf{s}_i\}$ to give

$$U = \frac{\kappa_B}{L}\sum_i^n \frac{L\left(1-\left(\mathbf{s}_i\cdot\mathbf{s}_{i+1}/|\mathbf{s}_i||\mathbf{s}_{i+1}|\right)^2\right)\left(|\mathbf{s}_i| + |\mathbf{s}_{i+1}|\right)}{|\mathbf{s}_i + \mathbf{s}_{i+1}|^2} + \frac{\kappa_S L^2}{2}\sum_i^n \left(\frac{|\mathbf{s}_i|}{L} - 1\right)^2,$$

written such that summation prefactors represent characteristic bending (κ_B/L) and stretching ($\kappa_S L^2/2$) energies. For nanoring configurations to be governed by bending (rather than stretching), $\kappa_S \gg 2\kappa_B/L^3$ is used. Bending limitations (imposed to prevent the overlapping of neighbouring, and distant, porphyrin macrocycles) are achieved by placing hard discs of diameter $\sqrt{3}L/2$ at the midpoints between bends. Overlapping of these discs results in an infinite energy penalty.
To generate thermally-equilibrated nanorings obeying by the energetics described above, Monte Carlo simulations are run using the Metropolis algorithm. The characteristic bending, κ_B/L, and

thermal, k_BT energies are the only relevant energy scales. The ratio of these defines a dimensionless rigidity $R = \kappa_B/Lk_BT$; the sole parameter controlling the energetics of the model.

The departure from circularity of a nanoring is characterised by a single parameter, g (see main text). To find an average nanoring non-circularity, $\bar{g}$, for a particular set of parameters, R and n, a nanoring is initially set in a circular configuration. The system evolves via the Metropolis algorithm and equilibrium is reached after an initial transient period. Equilibrium non-circularity values are recorded for an extensive time period, and a time-averaged value $\bar{g}$ can be obtained. For complete rigor, time-averaged values are obtained from many independent simulations (using different random seeds) and subsequently averaged. By correlating $\bar{g}$ values obtained from simulations with those measured from STM images, it was shown in Ref. 16 that $R \approx 6$ accurately reproduces nanoring configurations for both c-P12 and c-P24 nanorings adsorbed on an Au(111) surface at room temperature ($T = 290$ K), i.e. the bending rigidity κ_B (and porphyrin periodicity L) is inherently common to all rings.

Figure 17 shows how $\bar{g}$ varies with temperature (over the range $T = 220 - 360$ K) for five different ring sizes ($n = 8, 12, 16, 20, 24$) for dimensionless rigidities given by $R = 6 \times (290/T)$. For a given

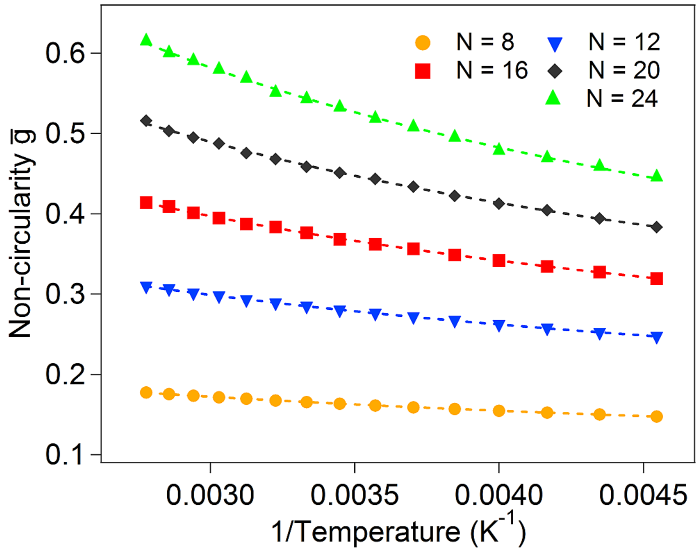

Figure 17: Ensemble-averaged non-circularity values plotted as a function of inverse temperature for five ring sizes. Non-circularity increases by increasing temperature and/or ring size. All lines are guides to the eye.

ring size, increasing temperature increases the average distortion from circularity of a nanoring. One can also see that for a given temperature, increasing ring size increases the average distortion from circularity (the five data points at $T = 290$ K are shown in Figure 3(c) of the main text).

References

1. O'Sullivan, M. C.; Sprafke, J. K.; Kondratuk, D. V.; Rinfray, C.; Claridge, T. D. W.; Saywell, A.; Blunt, M. O.; O'Shea, J. N.; Beton, P. H.; Malfois, M. et al. Vernier templating and synthesis of a 12-porphyrin nano-ring. *Nature* **2011**, *469*, 72–75.

2. Taylor, P. N.; Anderson, H. L. Cooperative self-assembly of double-strand conjugated porphyrin ladders. *J. Am. Chem. Soc.* **1999**, *121*, 11538–11545.

3. Saywell, A.; Sprafke, J. K.; Esdaile, L. J.; Britton, A. J.; Rienzo, A.; Anderson, H. L.; O'Shea, J. N.; Beton, P. H. Conformation and packing of porphyrin polymer chains deposited using electrospray on a gold surface. *Angew. Chem. Int. Ed.* **2010**, *49*, 9136–9139.

4. Sprafke, J. K.; Kondratuk, D. V.; Wykes, M.; Thompson, A. L.; Hoffmann, M.; Drevinskas, R.; Chen, W.; Yong, C. K.; Kärnbratt, J.; Bullock, J. E. et al. Belt-Shaped π-Systems: Relating Geometry to Electronic Structure in a Six-Porphyrin Nanoring. *J. Am. Chem. Soc.* **2011**, *133*, 17262–17273.

5. Kondratuk, D. V.; Perdigao, L. M. A.; O'Sullivan, M. C.; Svatek, S.; Smith, G.; Shea, J. N. O.; Beton, P. H.; Anderson, H. L. Two Vernier-templated routes to a 24-porphyrin nanoring. *Angew. Chem. Int. Ed.* **2012**, *51*, 6696–6699.

6. Hoffmann, M.; Kärnbratt, J.; Chang, M. H.; Herz, L. M.; Albinsson, B.; Anderson, H. L. Enhanced pi-conjugation around a porphyrin[6] nanoring. *Angew. Chem. Int. Edit. Engl.* **2008**, *120*, 5071.

7. Grozema, F. C.; Houarner-Rassin, C.; Prins, P.; Siebbeles, L. D. A.; Anderson, H. L.

Supramolecular control of charge transport in molecular wires. *J. Am. Chem. Soc.* **2007**, *129*, 13370–13371.

8. Drobizhev, M.; Stepanenko, Y.; Rebane, A.; Wilson, C. J.; Screen, T. E. O.; Anderson, H. L. Strong cooperative enhancement of two-photon absorption in double-strand conjugated porphyrin ladder arrays. *J. Am. Chem. Soc.* **2006**, *128*, 12432–12433.

9. Hoffmann, M.; Wilson, C. J.; Odell, B.; Anderson, H. L. Template-directed synthesis of a pi-conjugated porphyrin nanoring. *Angew. Chem. Int. Ed.* **2007**, *46*, 3122–3125.

10. Chang, M. H.; Hoffmann, M.; Anderson, H. L.; Herz, L. M. Dynamics of excited-state conformational relaxation and electronic delocalization in conjugated porphyrin oligomers. *J. Am. Chem. Soc.* **2008**, *130*, 10171–10178.

11. Valeur, B. *Molecular Fluorescence: Principles and Applications*; Wiley-VCH: Weinheim, 2002.

12. Mello, J. C. D.; Wittmann, H. F.; Friend, R. H. An improved experimental determination of external photoluminescence quantum efficiency. *Adv. Mater.* **1997**, *9*, 230–.

13. Kubin, R. F.; Fletcher, A. N. Fluorescence quantum yields of some rhodamine dyes. *J. Lumines.* **1982**, *27*, 455–462.

14. Rurack, K.; Spieles, M. Fluorescence quantum yields of a series of red and near-infrared dyes emitting at 600-1000 nm. *Anal. Chem.* **2011**, *83*, 1232–1242.

15. Duncan, T. V.; Susumu, K.; Sinks, L. E.; Therien, M. J. Exceptional near-infrared fluorescence quantum yields and excited-state absorptivity of highly conjugated porphyrin arrays. *J. Am. Chem. Soc.* **2006**, *128*, 9000–9001.

16. Svatek, S. A.; Perdigao, L. M. A.; Stannard, A.; Wieland, M. B.; Kondratuk, D. V.; Anderson, H. L.; O'Shea, J. N.; Beton, P. H. Mechanical stiffening of porphyrin nanorings through supramolecular columnar stacking. *Nano Lett.* **2013**, *13*, 3391–3395.
